# Non-targeted Plasma Metabolome of Early and Late Lactation Gilts

**DOI:** 10.3389/fmolb.2016.00077

**Published:** 2016-11-24

**Authors:** Lea A. Rempel, Jeremy R. Miles, William T. Oliver, Corey D. Broeckling

**Affiliations:** ^1^United States Department of Agriculture, Agricultural Research Service, U.S. Meat Animal Research CenterClay Center, NE, USA; ^2^Proteomics and Metabolomics Facility, Colorado State UniversityFort Collins, CO, USA

**Keywords:** GC-MS, lactation, LC-MS, non-targeted, pigs, plasma, metabolomics

## Abstract

Female pigs nursing their first litter (first-parity gilts) have increased energy requirements not only to support their piglets, but they themselves are still maturing. Non-targeted plasma metabolomics were used to investigate the differences between (1) post-farrowing and weaning (early or late lactation), (2) degree of body condition loss after lactation (extreme or minimal), and (3) interactions; to potentially identify compounds or pathways that could aide in alleviating energetic demands of lactation in gilts. Twenty first-parity gilts were selected with similar (*P* ≥ 0.4475) number of piglets born and nursed, and similar (*P* ≥ 0.3141) body condition traits (e.g., body weight and backfat thickness) post-farrowing, yet exhibited minimal or extreme loss (*P* ≤ 0.0094) in body weight (8.6 ± 1.48 kg and 26.1 ± 1.90 kg, respectively) and backfat thickness (1.3 ± 0.67 mm and 4.7 ± 0.86 mm, respectively) following lactation (weaning). Plasma samples from first-parity gilts at post-farrowing and weaning were investigated using UPLC-MS and GC-MS to generate a comprehensive metabolic profile. Each approach yielded approximately 700 detected features. An ANOVA was performed on each detected compound in R for time of collection, body condition change, and the interaction, followed by a false discovery correction. Two unknown features were different (*P* ≤ 0.05) for extreme vs. minimal body condition change. Several compound differences (*P* ≤ 0.05) were identified between post-farrowing and weaning. Thirty-two features detected by UPLC-MS had at least a log_2_ fold-change of ±1.0 while only 18 features had a log_2_ fold-change of ±0.6 or more for the significant GC-MS features. Annotation implicated various metabolic pathways. Creatinine was greater at weaning (*P* = 0.0224) and others have reported increased serum concentrations of creatinine in response to body weight loss. Hippurate and caprolactam, associated with protein catabolism, were also greater (*P* ≤ 0.0166) at weaning. Phospholipid features (*P* ≤ 0.0347) and inositol-related features (*P* ≤ 0.0236) were also greater at weaning. Inositol features may exert insulin-like effects. The energetic demands of lactation in gilts nursing their first litter indicated a greater difference exists between early and late lactation regardless of body condition loss.

## Introduction

Giving birth to and raising a litter of piglets is a very energy-demanding event. First-parity gilts, sexually mature females giving birth to their first litter of piglets, are not only vulnerable to the stress of lactation, but also supporting their own continued physical maturation, which typically is complete just prior to their second litter of piglets (refer to Table 1 for common terms and definitions). Excessive weight loss during lactation reflects tissue catabolism in exchange for mammary metabolic demands and can have subsequent adverse effects on reproductive parameters such as weaning-to-estrus interval, number of days until sexual receptivity following weaning (Reese et al., 1984; Serenius et al., 2006). Lactation weight loss includes both lipid and muscle catabolism (O'grady et al., 1975; Duee and Desmoulin, 1982; Clowes et al., 2005; Rempel et al., 2015), and post-weaning recovery of muscle is less robust in first-parity gilts in comparison to mature sows (Rempel et al., 2015).

**Table 1:** **List of commonly used abbreviations and terms**.

**Abbreviation or term**	**Meaning**
BFT	Backfat thickness
BW	Body weight
CSUPMF	Colorado State University Proteomics and Metabolomics Facility
Dam	Female that has progeny or offspring.
Hi Loss	Sows with extensive body weight and backfat thickness loss at weaning
Lactation	Period of milk production following farrowing in which young receive nutrition by suckling/nursing
LEA	Loin Eye Muscle Area
Lo Loss	Sows with minimal body weight and backfat thickness loss at weaning
NBA	Number Born Alive; number of piglets alive at birth
Parity	Farrowing event for a female pig
PC	Principal component
PCA	Principal component analysis
PF	Post-Farrowing; following a farrowing event
Sow	Female pig that has farrowed at least one litter of piglets
TNB	Total Number Born; number of total piglets born in the litter
WN	Weaning; removal of piglets from a nursing sow
WND	Number Weaned; number of piglets weaned in the litter
Weaning-to-Estrus Interval	The number of days following weaning until a female becomes receptive for breeding.

“Thin Sow Syndrome” was a phrase coined several years ago to describe a visible loss of condition; whereas, more recently “Shattered Sow Syndrome” was proposed to describe a more severe (16–18%) protein loss during lactation (Gadd, 2009). A “shattered” sow would be under extreme metabolic duress at the time of weaning, greatly reducing fertility and lifetime production. In contrast to the shattered sow, the swine industry is increasingly concerned about sows that are systematically depleted through catabolism and bone density loss over several parities, eventually compromising animal welfare, fertility, and lifetime productivity. Similar effects and reduced fertility have been commonly seen in high-producing dairy cattle (Lucy, 2001; Wathes et al., 2007). Management strategies during late lactation and post-lactation to improve reproductive performance in dairy cattle have included combinations of genotype, nutrition, immune function, and/or metabolic processes (Lucy, 2001; Gutierrez et al., 2006). Several individual trait studies have been performed on swine. However, a more complete interpretation of dam biochemistry during lactation will allow us to discover changes related to phase of lactation and concomitant weight loss that could be modified through diet or management strategies.

Thus, the objective of the current study was to measure the plasma metabolome of first-parity gilts at early and late lactation with divergent body condition loss at weaning (minimal or extreme) to evaluate the influence of the main effects and their interaction. Our results suggest that time of lactation has a greater influence on changes in plasma biochemistry in contrast to body condition changes or the interaction of lactation phase and body condition loss.

## Materials and methods

All experimental procedures and techniques were reviewed and approved by the U.S. Meat Animal Research Center (USMARC) Institutional Animal Care and Use Committee (EO3040-31000-091-04). Procedures for handling animals complied with the Guide for the Care and Use of Agricultural Animals in Research and Teaching (FASS, 2010).

### Animal and sample collection

Landrace-Duroc-Yorkshire dams at USMARC entered the farrowing facility at approximately 110 days (d) gestation and received a standard 19% crude protein lactation diet of 2.0 kg per day that met or exceeded NRC recommendations (National Research Council, 2012). On the day of farrowing dams did not receive feed. On d1 post-farrowing (PF) sows were fed 2.7 kg, were allowed to consume feed *ad libitum* from d2 PF through weaning (WN). Over the course of 12 weekly farrowing groups, 68 first parity dams were processed for the following measurements; d1 PF and WN body weight (BW), PF and WN 10th rib backfat thickness (BFT), PF and WN 10th rib loin eye area muscle (LEA), total number of piglets born (TNB), total number of piglets born alive (NBA), and number of piglets weaned (WND). Ultrasound measurements were conducted twice a week at an average of; 2.7 ± 1.45 d lactation for PF and 26.7 ± 1.38 d lactation for WN. Tenth rib BFT and LEA were imaged using an Aloka 500SD ultrasound with a UST-5011-3.5 linear transducer (Hitachi Aloka Medical America, Inc., Wallingford, CT, USA). Measurements for BFT and LEA were conducted in duplicate using the Bioquant Nova Prime Image system (v.6.9.1, BIOQUANT Image Analysis Corp., Nashville, TN, USA). Reductions in BW, BFT, and LEA were derived by calculating the change from PF measurement to WN measurement.

Blood samples were collected using a non-surgical marginal ear vein technique PF and just prior to WN on the same day as ultrasound measurements between 0730 and 0800. The base of the ear was occluded and approximately 2.5 ml blood was collected using a 21 gauge infusion set into an S-Monovette tube (Sarstedt, Newton, NC, USA) containing 100 uL 4% ethylenediaminetetraacetic acid. Plasma was separated using centrifugation at 2000 × g, 4°C for 20 min. Two hundred uL of plasma was stored at −80°C until extraction and analyses were performed.

**Figure 1:**
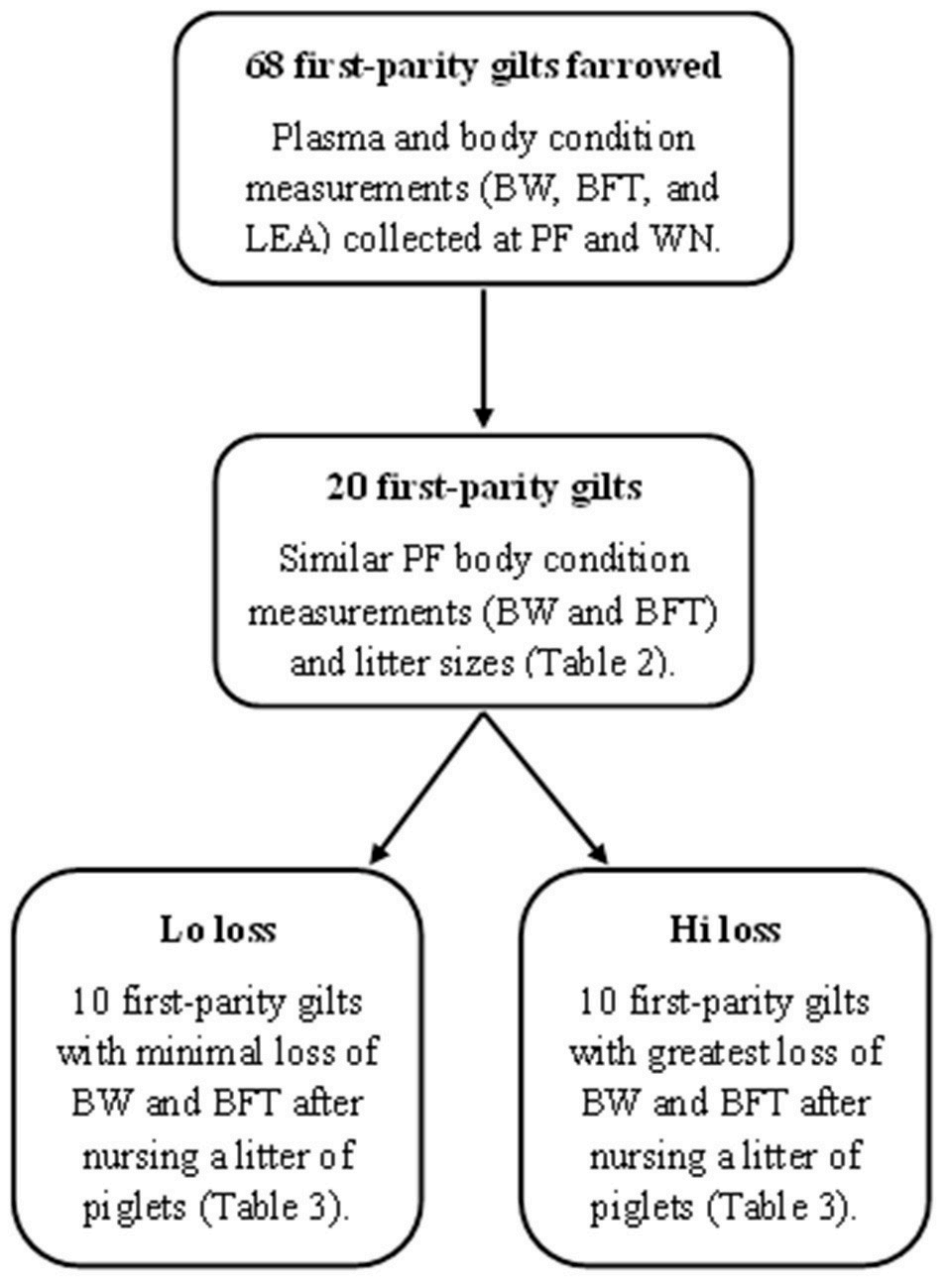
**Diagram of methods used to select animals for non-targeted plasma metabolome analysis (GC-MS and LC-MS) from early and late lactation**. Post-farrowing (PF), weaning (WN), body weight (BW), backfat thickness (BFT), and loin eye area muscle (LEA).

From the 68 first parity dams sampled, 20 were retrospectively identified as having similar (*P* ≥ 0.10) PF body condition measurements and litter sizes (Table 2). From these dams, 10 dams with the least change (Lo loss) and 10 dams with the greatest reduction (Hi loss) in BW and BFT from PF to WN (Table 3) had PF and WN plasma samples submitted for non-targeted metabolomics analysis at Colorado State University Proteomics and Metabolomics Facility (CSUPMF). The approach for animal selection is also presented as an illustration (Figure 1).

**Table 2:** **Age, body condition, and litter traits (LSM ± SE) among first parity females were similar (***P*** ≥ 0.10)**.

**Variable**	***P*-value**	**Lo Loss**	**Hi Loss**
Farrowing age (d)	0.8914	343.3±4.47	342.3±6.33
d1 PF BW (kg)	0.7678	183.7±4.16	185.8±6.02
PF 10th rib backfat thickness (BFT; mm)	0.8574	19.9±0.94	19.6±1.37
PF 10th rib loin eye area (LEA; cm^2^)	0.3278	43.6±1.79	46.8±2.61
Total number born (TNB)	0.4304	12.9±0.74	11.8±1.08
Number born alive (NBA)	0.7231	12.7±0.87	12.4±0.97
Number weaned (WND)	0.8395	10.3±0.37	10.5±0.54

*LSM ± SE, least-square mean ± standard error; BW, body weight; BFT, 10th rib backfat thickness; LEA, 10th rib loin eye area; TNB, total number born; NBA, number born alive; WND, number weaned*.

**Table 3:** **Loss of BW, BFT, and LEA (LSM ± SE) in dams with the least decrease (Lo Loss; ***n*** = 10) or greatest decrease (Hi Loss; ***n*** = 10) over the course of lactation**.

**Variable**	***P*-value**	**Lo Loss**	**Hi Loss**
BW Loss (kg)	<0.0001	10.6 ± 1.32	27.5 ± 1.90
BFT Loss (mm)	0.0269	2.5 ± 0.66	5.4 ± 0.94
LEA Loss (cm^2^)	0.3748	4.3 ± 1.05	6.0 ± 1.52

*LSM ± SE, least-square mean ± standard error; BW, body weight; BFT, 10th rib backfat thickness; LEA, 10th rib loin eye area*.

### Non-targeted metabolome profiling

Plasma samples were thawed at 4°C, and 100 uL was transferred to a 650 uL 96-well plate containing 400 uL of cold methanol per well. The mixture was centrifuged at 3000 × g for 20 min at 4°C, and the supernatant was aliquoted into two separate 96-well plates; 200 uL for GC-MS analysis, 200 uL for UPLC-MS analysis.

For GC-MS, 200 μL of extract was dried using a speedvac, resuspended in 50 μL of pyridine containing 15 mg/mL of methoxyamine hydrochloride, incubated at 60°C for 45 min, sonicated for 10 min, and incubated for an additional 45 min at 60°C. Next, 50 μL of N-methyl-N-trimethylsilyltrifluoroacetamide with 1% trimethylchlorosilane (Thermo Scientific, Waltham, MA, USA) was added and samples were incubated at 60°C for 30 min, centrifuged at 3000 × g for 5 min, cooled to room temperature, and 80 μL of the supernatant was transferred to a 150 μL glass insert in a GC-MS autosampler vial. Metabolites were detected using a Trace GC Ultra coupled to a Thermo ISQ mass spectrometer (Thermo Scientific). Samples were injected in a 1:10 split ratio twice in discrete randomized blocks. Separation occurred using a 30 m TG-5MS column (Thermo Scientific, 0.25 mm i.d., 0.25 μm film thickness) with a 1.2 mL/min helium gas flow rate, and the program consisted of 80°C for 30 sec, a ramp of 15°C per min to 330°C, and an 8 min hold. Masses between 50 and 650 m/z were scanned at 5 scans/s after electron impact ionization.

**Figure 2:**
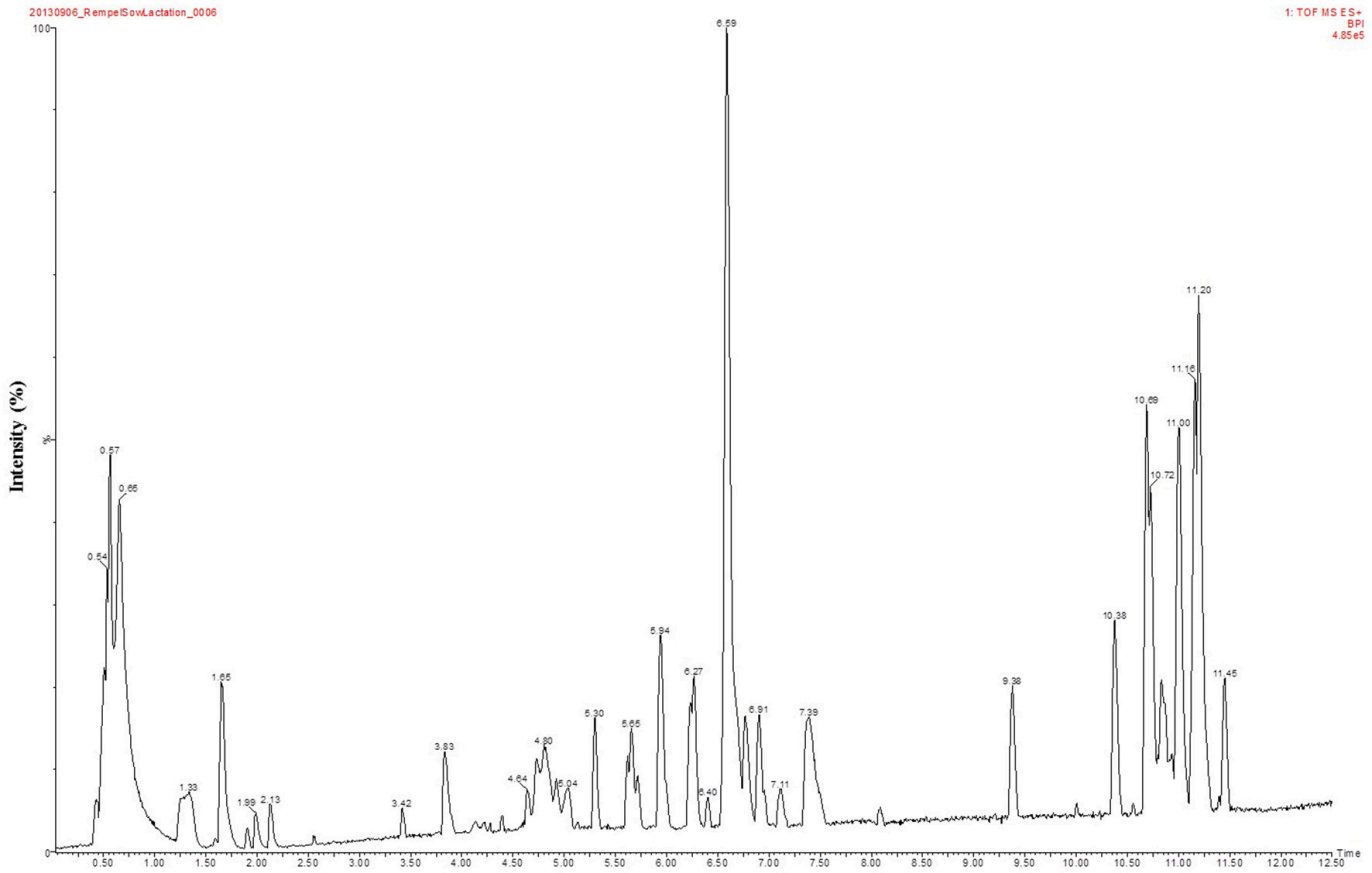
**A representative LC-MS base peak chromatogram of plasma collected at post-farrowing from a dam that had minimal body condition loss at the conclusion of lactation (Lo loss)**. The Y-axis represents the intensity (%) and the X-axis depicts retention time in minutes.

For UPLC-MS analysis, 1 uL of metabolite extract was injected twice (*n* = 2 replicates) onto a Waters Acquity UPLC system (Waters Corp., Milford, MA, USA) in discrete, randomized blocks, and separated on a Waters Acquity UPLC C8 column (1.8 μM, 1.0 × 100 mm), using a gradient from solvent A (95% water, 5% methanol, 0.1% formic acid) to solvent B (95% methanol, 5% water, 0.1% formic acid). Injections were made in 100% A, which was held for 0.1 min, ramped to 40% B in 0.9 min, to 70% B over 2 min, and to 100% B over 8 min. Mobile phase was held at 100% B for 6 min, returned to starting conditions over 0.1 min, and allowed to re-equilibrate for 5.9 min. Flow rate was constant at 140 μL/min for the duration of the run. The column was held at 50°C, samples were held at 10°C. The column eluent was infused into a Waters Xevo G2 Q-TOF-MS (Waters Corp.) with an electrospray source in positive mode, scanning 50–1200 m/z at 0.2 s per scan, alternating between MS (6 V collision energy) and MSE mode (15–30 V ramp). Calibration was performed using sodium formate with 1 ppm mass accuracy. The capillary voltage was held at 2200 V, source temp at 150°C, and nitrogen desolvation temperature at 350°C with a flow rate of 800 L/h. A base peak chromatogram from a representative sample is provided in Figure 2.

### Analyses

#### Animal production statistics

Physiological data were analyzed using the Mixed procedure of SAS (SAS Institute, Cary, NC, USA). The model used to derive the least square means for farrowing age, total number born (TNB), number born alive (NBA), number weaned (WND), BW, BFT, and LEA included the discrete effects of farrowing group (*n* = 9) and body condition group (Lo loss or Hi Loss) with the random effect of sire (*n* = 11). All data were collected in 2013; therefore, year was excluded from the model.

#### Feature detection and alignment

For each sample, raw data files were converted to CDF format, and a matrix of molecular features, as defined by retention time and mass (m/z), was generated using XCMS software in R (Smith et al., 2006; R Development Core Team, 2013). Raw peak areas were normalized to total ion signal in R, outlier injections were detected based on total ion signal and PC1 of principle component analysis (PCA), and the mean area of the chromatographic peak was calculated among replicate injections (*n* = 2). Features were grouped into spectra, based on coelution and covariance parameters across the full dataset using RAMClustR (Broeckling et al., 2014). Breifly, RAMClustR groups several features derived from one compound (e.g., isotopes, adducts, fragments, multimers, etc) into a single unit, reducing analytical variability, then reconstructed the spectra so it was suitable for spectral metabolite database searches.

#### ANOVA and principal component statistics

Analysis of variance was conducted on each derived feature using the aov function in R, and false positives were controlled using the Benjamini-Hochberg method in the p.adjust function in R (Benjamini and Hochberg, 1995) with a significance level of 0.05. Principal component analysis was conducted on mean-centered and Pareto variance-scaled data using the pcaMethods package in R.

#### Metabolomic annotation

Features were annotated based on spectral matching, electron ionization spectra for GC-MS and in-source MS and idMS/MS for LC-MS data, to CSUPMF, NISTv12, Golm (GC-MS only), Metlin, and Massbank metabolite databases. Each proposed annotation was verified by MS/MS fragmentation analysis of the feature in comparison to, (1) MS/MS fragmentation of the putative metabolite, and/or (2) mass spectral database, and/or (3) from a commercially available pure standard (Sigma-Aldrich, St. Louis, MO, USA). All features were scored from 1 to 4 for identification level as per the Metabolomics Standards Initiative; 1—both experimental mass spectrum and RT match an authentic standard ran in-house under the same conditions (CSUPMF library); 2—experimental mass spectrum matches an authentic standard; 3—experimental mass spectrum indicates a specific chemical class; and 4—experimental mass spectrum does not match any class or standard in the MS databases (Sumner et al., 2007).

## Results

Of the original 68 first parity dams, 20 were identified as having similar (*P* ≥ 0.10) PF body condition traits (BW, BFT, LEA) as well as similar (*P* ≥ 0.10) litter sizes (TNB, NBA, WND; Table 2). However, by WN, these females had differing (*P* ≤ 0.05) BW and BFT loss (Table 3). Plasma samples from PF and WN were submitted for non-targeted metabolomics to determine if biomarkers were present that could help define the physiological pathways influenced during lactation. Following WN, five of the Hi loss females failed to express estrus and were not bred and a sixth Hi loss female was bred within 5 days post-WN, but failed to maintain her second pregnancy to term. Whereas all Lo loss females were rebred following weaning, and only two failed to maintain their second pregnancy to term.

**Figure 3:**
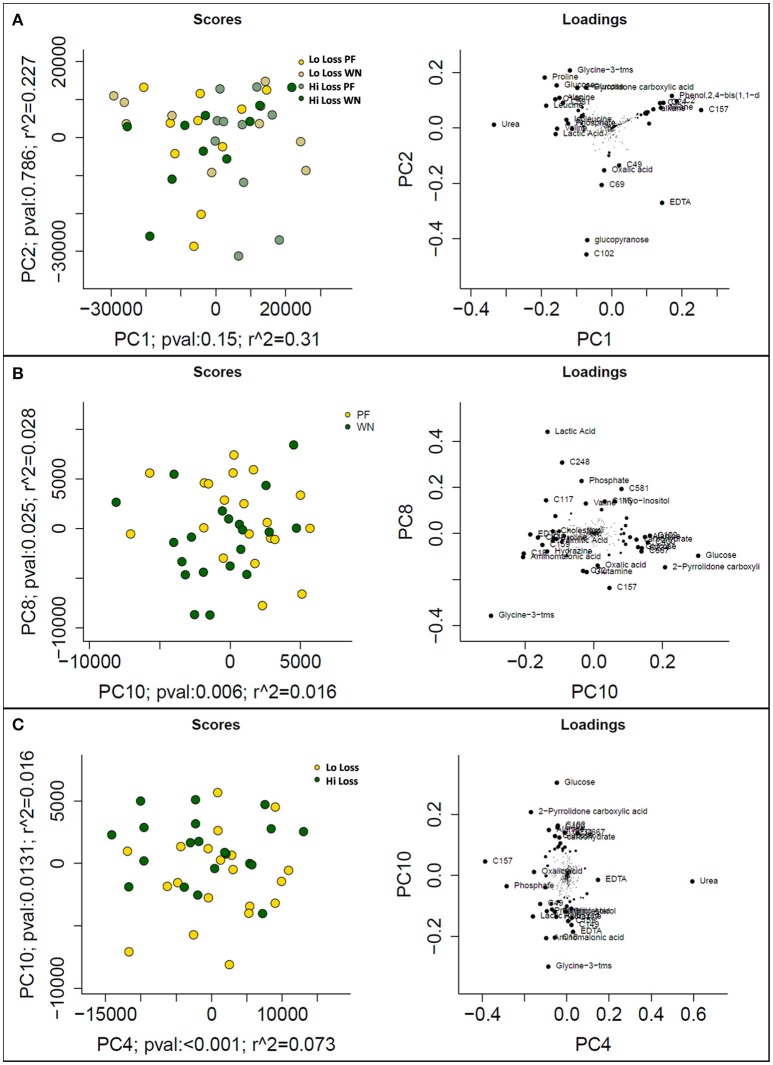
**Prinicipal component analysis scores (left) and loadings (right) plots from Pareto scaled GC-MS dataset. (A)** displays the maximal explained variance, plotting PCs 1 and 2, and shows no distinguishable pattern for time of sampling or body condition of dam. **(B)** plots optimal separation based on time of sampling (post-farrowing or weaning) after correcting for subject differences, and **(C)** plots optimal separation by body condition of dams (lo loss and hi loss) following adjustment for subject differences.

Following data acquisition and feature detection and alignment, 770 and 692 metabolic features from sow plasma were detected using GC-MS and LC-MS techniques, respectively. Innately, GC- and LC- methods detect different compounds (derivatized volatile organic compounds vs. liquid polar compounds, respectively) thereby providing a more diverse metabolome profile. Principal component analysis of the GC-MS dataset revealed that experimental factors were not the primary sources of variation in the plasma metabolome (PF vs. WN, Lo loss vs. Hi loss; Figure 3A). However, higher level principle components, which explain variation orthogonal to that of PC1 do suggest that the metabolome is responsive to time and weight loss. For example, PCs 8 and 10 collectively explain 4.4% of the Pareto scaled dataset wide variation and partially separated based on time of sampling (PF and WN; Figure 3B) and PCs 4 and 10 explain 8.9% of the Pareto scaled dataset wide variation partially separated based on body condition (Lo loss and Hi loss; Figure 3C). The loading plots featured to the right of the PC plots indicate those features that contributed most to PCA results (Figure 3). Increased concentrations of urea pull Figure 3A toward the upper right. Phosphate, lactic acid, and a non-annotated compound (C248) push the PF samples toward the upper right in Figure 3B. The loading plot for PC10 vs. PC4 is (Figure 3C) suggests glucose pulls the Hi Loss samples toward the upper left whilst glycine and urea push the Lo Loss samples toward the bottom right.

**Figure 4:**
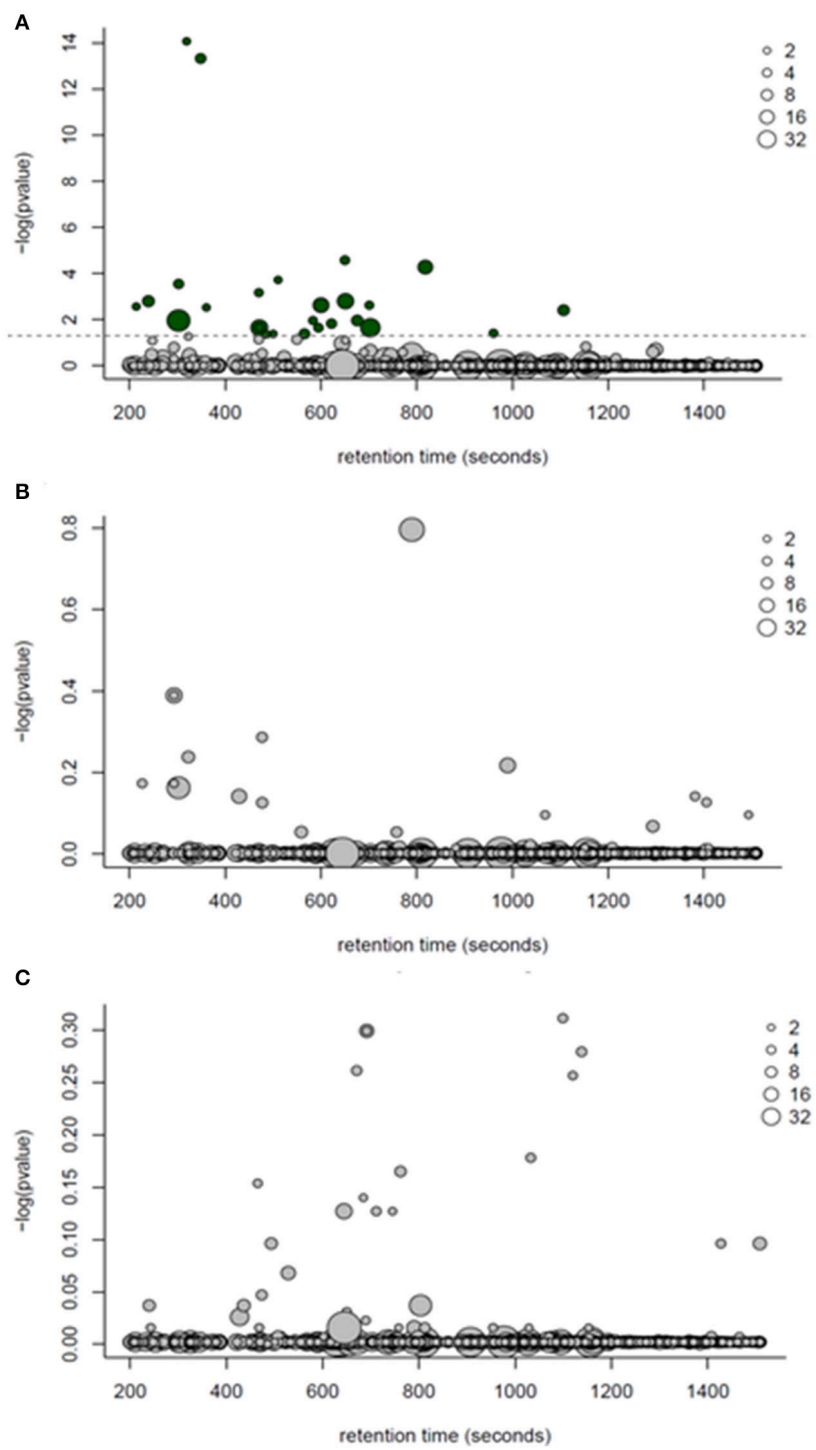
**ANOVA bubble plots for GC-MS detected features. (A)** *p*-values in this panel represent the ANOVA main effect for time (post farrow vs. weaning). **(B)** represents the separation of features by body condition loss over lactation (hi loss vs. lo loss). **(C)** depicts the interaction of time and body condition loss. Dotted line represents significance (*P* < 0.05) threshold following Benjamini-Hochburg adjustment. Size of points represents the number of features detected and dark bubbles represent those which are significant (*P* < 0.05).

One-way ANOVA tests followed by a Benjamini-Hochburg false discovery rate correction were applied to each of the 770 features detected by GC-MS; the results identified several features which differed between PF and WN (*P* < 0.05; Figure 4A), whilst no differences (*P* > 0.05) were detected between Lo loss and High loss or the interactions (Figures 4B,C). Twenty-seven annotated and unknown features were different (*P* ≤ 0.05) between PF and WN. Annotated features are reported in Table 4 along with log_2_ fold-change. Putatively identified metabolites that were greater (*P* ≤ 0.05) at WN in contrast to PF included: inositol, tolyl-glucuronide, pinitol, creatinine, and urea. Only one metabolite greater (*P* ≤ 0.05) at PF vs. WN was annotated; myo-inositol. Twenty of the significant GC-MS detected metabolites remained unknown and are presented within the complete list of the GC-MS features (Supplementary Table 1).

**Table 4:** **Log_**2**_ fold-change (FC) differences between post-farrow and weaning following ANOVA analyses (***P*** ≤ 0.05), among detected features by GC-MS**.

**Annotated compound**	**FC**	***p*-value**	**Score^a^**	**RT (s)**	**Mass**
Inositol	−1.35	0.0016	2	651.15	612
Tolyl-glucuronide	−1.22	<0.0001	2	818.02	572
Pinitol	−1.04	0.0024	2	599.98	554
Pinitol	−1.00	0.0024	2	600.07	554
Creatinine	−0.66	0.0224	2	471.20	329
Urea	−0.44	0.0109	2	302.22	204
Myo-Inositol	0.68	0.0224	2	703.08	612

*FC, log_2_ fold-change; PF, post-farrow; WN, weaning; ANOVA, analysis of variance; GC-MS, gas chromatography-mass spectrometry; RT, retention time*.

a *Annotation confidence score (scale of 1–4) based on guidelines provided by the Metabolomics Standards Initiative (Sumner et al., 2007)*.

**Figure 5:**
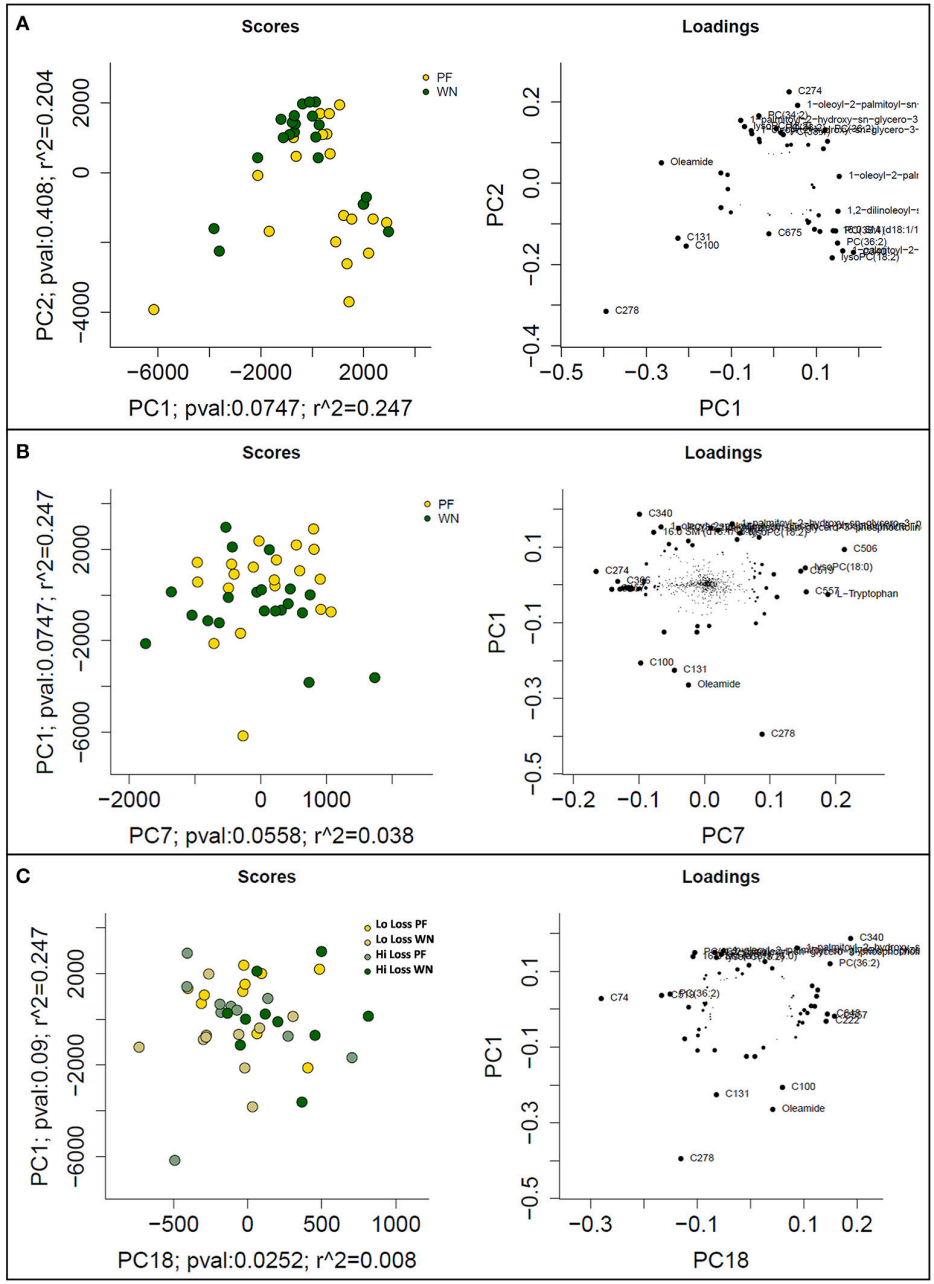
**PCA score plots (left) and loadings plots (right) from mean-centered and Pareto scaled LC-MS dataset. (A)** depicts the maximal variance, plotting PC1 vs. PC2, **(B)** represents optimal separation based on time of collection (post-farrowing or weaning), and **(C)** represents optimal separation by body condition loss (lo loss or hi loss) with respect to time of sampling (post-farrowing or weaning).

Six hundred ninety-two features were detected in sow plasma using LC-MS techniques. The collective results from PCs 1 (PF vs. WN) and 2 (Lo loss vs. Hi loss) explain ~41% of the variation observed in the Pareto scaled data (Figure 5A). The complimentary loading plot suggested compound C278 influenced PF samples whilst features C100 and C131 pushed weaning samples toward the left. To further evaluate the influence of timing of sample collection (PF vs. WN), PC comparisons for PC1 and PC7 suggest a marginal (*P* = 0.075) contribution of separation was due to time of sampling (Figure 5B). The companion loadings plot indicated a strong influence from forms of phosphatidyl choline lipids, located in the upper portion of the plot (labeled features that are overlapping each other; Figure 5B). Separation of features by body condition (Lo loss vs. Hi loss) alone was incomplete; therefore, PCA plots separated by body condition were performed with respect to time of sampling as an interaction cofactor (Figure 5C). The loadings plot indicated a lower left to upper right separation with C278, C131, C340, and several phosphatidylcholines contributing the most to this distribution of features (Figure 5C).

**Figure 6:**
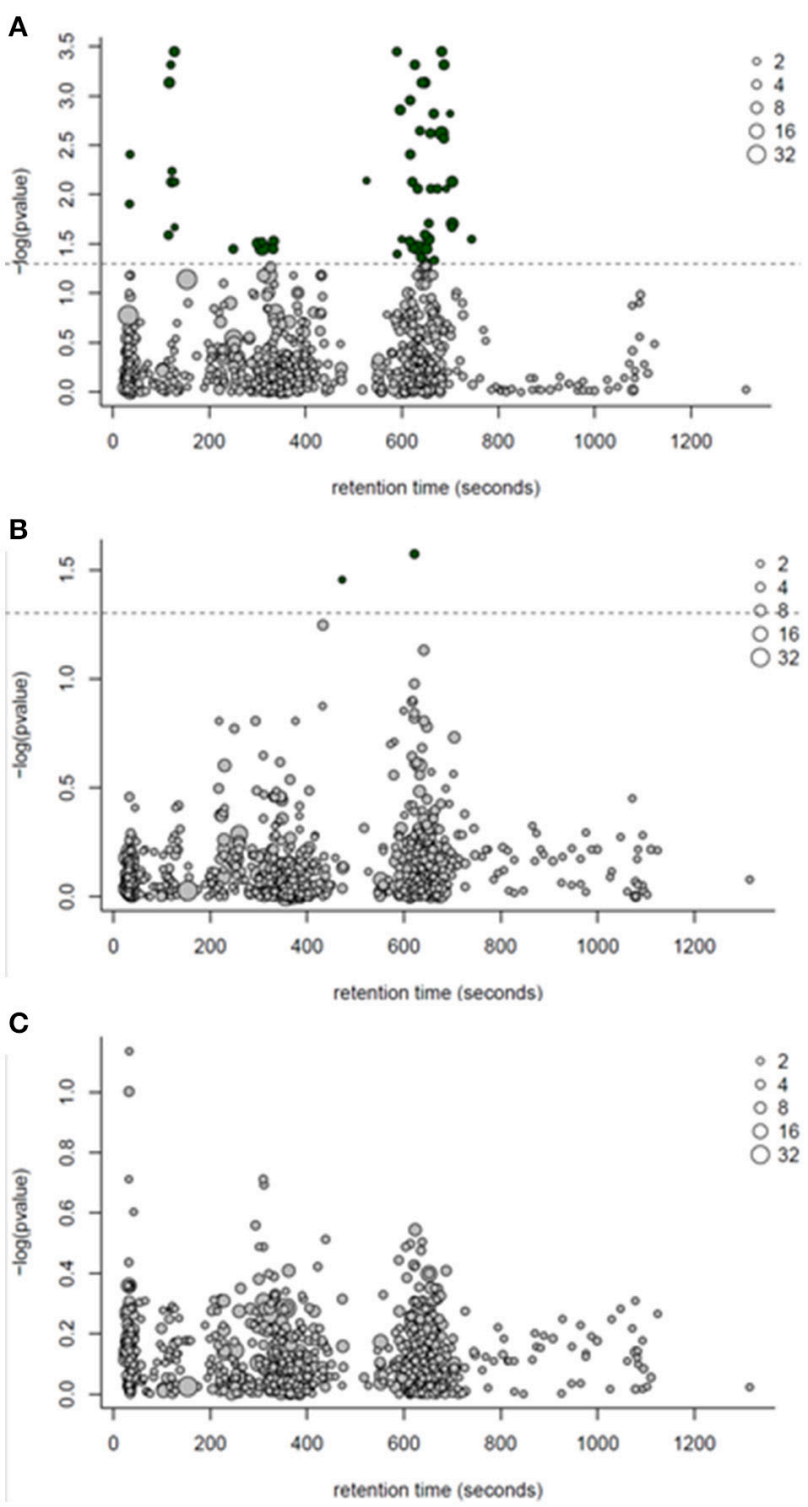
**ANOVA bubble plot for LC-MS detected features. (A)** represents features separating by the effect of time (post-farrow vs. weaning). **(B)** represents the separation of features by lactation body condition loss (hi loss vs. lo loss). **(C)** depicts the interaction of time and body condition loss. Dotted line represents threshold for significance (*P* < 0.05) following a Benjamini-Hochburg adjustment. Size of points represents the number of features detected and dark bubbles represent those which are significant (*P* < 0.05).

Following one-way ANOVA with a false discovery rate correction of the LC-MS data, 54 compounds or features were significant (*P* ≤ 0.05) between PF and WN (Figure 6A). Sixteen of the 54 significant features were annotated and are reported in Table 5, whilst only two features were different (*P* ≤ 0.05) between Lo loss and Hi loss samples (Figure 6B and Table 6). No differences (*P* > 0.05) were determined when features were tested for the interaction of lactation phase by body condition loss (Figure 6C). Annotation resulted in 54 metabolites with a confidence score of 1 and included compounds such as; caprolactam, phosphatidylcholine, hippuric acid, L-tryptophan, betaine, and cholic acid. All 692 LC-MS detected compounds or features are provided as a Supplementary data (Supplementary Table 2).

**Table 5:** **Log_**2**_ fold-change (FC) differences between post-farrow and weaning following ANOVA analyses (***P*** ≤ 0.05), among detected compounds or features by LC-MS**.

**Annotated compound**	**FC**	***p*-value**	**Score^a^**	**RT (s)**	**Mass**
Caprolactam	−3.65	0.0257	1	115.0588	113.084
Phenylacetylglycine	−1.74	0.0058	2	122.3187	193.074
1,2-dioleoyl-sn-glycero-3-phosphoethanolamine	−1.54	0.0011	1	616.9482	743.547
PC(36:2).2	−1.29	0.0007	2	647.313	772.5867
Hippuric acid	−1.28	0.0007	1	116.7797	179.058
lysoPC(18:2).2	−1.13	0.0311	2	300.192	520.3399
lysoPC(18:2)	−0.95	0.0347	2	309.5405	520.3402
C16-20:3 PC	−0.73	0.0024	1	659.0428	769.599
PC(36:3).3	−0.73	0.0255	2	648.0042	784.5853
PC(36:3)	−0.64	0.0007	2	641.6088	784.5871
Betaine	−0.64	0.0125	1	34.17233	117.079
PC(32:2)	−0.60	0.0345	2	622.7085	732.5558
1,2-dilinolenoyl-sn-glycero-3-phosphocholine	−0.56	0.0343	1	632.0548	777.531
PC(O-34:3)	−0.53	0.0356	2	650.9358	742.5767
SM(24:1)	0.82	0.0197	3	704.4908	813.6867
PC(40:4)	1.01	0.0024	2	682.2406	838.6347

*FC, log_2_ fold-change; PF, post-farrow; WN, weaning; ANOVA, analysis of variance; LC-MS, liquid chromatography-mass spectrometry; m/z, mass value of compound or base peak; PC, phosphatidylcholine; SM, sphingomyelin*.

a *Annotation confidence score (scale of 1–4) based on guidelines provided by the Metabolomics Standards Initiative (Sumner et al., 2007)*.

**Table 6:** **Log_**2**_ fold-change (FC) differences between maximal (Hi) and minimal (Lo) body condition loss following ANOVA analyses (***P*** ≤ 0.05), among detected features by LC-MS**.

**Compound identifier^a^**	**FC (Hi vs. Lo)**	***p*-value (Hi vs. Lo)**	**Score^b^**	**RT (s)**	**Mass**
C345	−0.77	0.0267	4	621.778	599.5044
C487	1.35	0.0350	4	472.369	203.0535

*FC, log_2_ fold-change; PF, post-farrow; WN, weaning; ANOVA, analysis of variance; LC-MS, liquid chromatography-mass spectrometry*.

a *Unidentified feature denoted as C###*.

b *Annotation confidence score (scale of 1–4) based on guidelines provided by the Metabolomics Standards Initiative (Sumner et al., 2007)*.

## Discussion

In the current study, we detected 770 and 692 features by GC-MS and LC-MS, respectively. These data seem very high, but it is highly probable that several features are actually replicates of the same compound detected as derivatized isoforms. We are confident in the number of detected features in the current study, which were derived from nearly 1.7 million peaks clustered into approximately 1400 features using XCMS software. As part of a training procedure within our lab, the same raw LC-MS data was processed using the XCMS program yielding a similar result (1640 features). Furthermore, the m/z scanning window was set from 50 to 1200 m/z allowing for a very broad spectral range.

Two possible features were found due to differences in body composition at weaning following false discovery corrections. However, they were unable to be annotated. Differences in plasma metabolomic profiles were evident between post-farrowing and weaning sampling periods. A plasma metabolomics study performed on transition sows using LC-MS techniques reported a weak separation between post-farrowing days 3 and 17 (Hedemann et al., 2012), similar to the principle component results within the current study for both GC-MS and LC-MS. Importantly, several of the putative compounds identified by database search were significantly different by sampling time (post-farrow vs. weaning). And a majority of these annotated products have direct or indirect relationships to energy metabolism. Interestingly, a few of the compounds may also indirectly affect reproductive ability and performance.

The identified compound, caprolactam, was much greater at weaning than post-farrowing. Caprolactam is most commonly known as a highly efficient precursor to commercial synthesis of L-lysine through enantiomer-selective hydrolysis (Sifniades et al., 1976). Caprolactam is a lactam of caproic acid (aka hexanoic acid; PubChem Compound Database, CID = 8892; Kim et al., 2016), which is a saturated medium chain fatty acid derived from hexane and found in plants and animal fats. Interestingly, caprolactam is a potent anti-calcitonin gene-related peptide (CGRP) receptor antagonist (Shaw et al., 2007). Calcitonin gene-related peptide, classified as a neuropeptide, has both metabolic as well as reproductive effects. In the rat, elevated levels of plasma CGRP were observed prior to the onset of obesity (Gram et al., 2005). It was recently reported that CGRP treatment down-regulated proteolytic activity in rat skeletal muscle by increasing cAMP and PKA/CREB signaling, inhibiting Foxo activity and LC3 lipidation (Machado et al., 2016). The metabolic action of caprolactam in our study was likely counter to the energetic actions of CGRP thereby allowing for increased tissue mobilization and/or reduced lipidation and protein accretion as lactation persisted. Interestingly, CGRP can also influence reproduction. Treatment of sexually mature gilts with chronic estradiol-17β reduced CGRP-immunoreactive ganglia innervating the ovary (Jana et al., 2012). Granulosa cells also express CGRP receptors and exogenous treatment of granulosa cell cultures with CGRP increased the production of testosterone and estradiol (Zhang et al., 2012). In mouse uteri, CGRP relaxes myometrium independent of the NOS pathway (Naghashpour and Dahl, 2000). Furthermore, CGRP varies throughout the estrous cycle and at estrus; the murine myometrium becomes less responsive to CGRP, likely increasing the contractile activity of the uterus improving sperm transport. In the current study, the increased levels of caprolactam may also be acting to prime or facilitate the uterine and ovarian response to CGRP for upcoming post-weaning reproductive activity.

The current study also identified increased levels of 1,2-dioleoyl-sn-glycero-3-phosphoethanolamine and putatively identified multiple forms of phosphatidylcholine (e.g., lysophospholipid, 1-oleoyl-2-palmitoyl-sn-glycero-3-phosphocholine, 1-stearoyl-2-hydroxy-sn-glycero-3-phosphocholine, 1-palmitoyl-2-hydroxy-sn-glycero-3-phosphocholine) at weaning. Phosphatidylcholine is necessary for lipoprotein formation and secretion (Cole et al., 2012). Levels of phosphatidylcholine are strongly associated with lipoprotein formation and impaired phosphatidylcholine production leads to hepatic uptake of low- and high- density lipoproteins from circulation. Impairment of lipoprotein metabolism can have non-hepatic downstream effects, including disrupted ovarian steroid hormone production (Grummer and Carroll, 1988). Treatment of porcine corpora lutea with phosphatidylcholine and phosphoethanolamine affected the fluidity of the plasma membrane thereby changing the accessibility of the porcine LH receptor (Scsukova and Kolena, 1995). In contrast to the general phosphatidylcholine levels, two sphingomyelins [18:0 SM and SM(24:1)] and one phosphatidylcholine isoform [PC(40:4)] were reduced at weaning in dams from the current study. Overweight humans consuming an energy-restricted diet experienced weight loss, decreased levels of sphingomyelins, and ameliorated metabolic syndrome (Martinez-Ramirez et al., 2016). Additionally sphingolipids, which include sphingomyelins, are known to influence steroid hormone production by regulating components of the biosynthesis pathway (Lucki and Sewer, 2008). Alterations to the lipid compounds between early lactation and just prior to weaning not only are likely a response to the changing body condition as a result of parturition and lactation, but may also be functioning to assist with post-weaning reproductive performance at the level of the ovary and steroid production and responsiveness.

Betaine was modestly increased at weaning in comparison to post-farrowing. These data were similar to the report from Hedemann et al. (2012), in which plasma betaine increased from day 3 to day 17 post-farrowing in sows similarly, cholic acid did not change by day of lactation but rather was different at site of collection (portal, hepatic, or arterial). Betaine is involved in hepatic protein and fat metabolism (Craig, 2004). Furthermore, as observed in the current experiment, increased betaine corresponds with increased phosphatidylcholine levels (Olthof and Verhoef, 2005). Thus, in the current study increased levels of betaine at weaning reflect the active hepatic protein and lipid metabolism necessary for energy homeostasis.

Marginally increased levels of creatinine were observed at weaning in the current study. Creatinine is the breakdown product of creatine, which is necessary for cellular energetics especially within skeletal muscle (Wyss and Kaddurah-Daouk, 2000) and may play a critical role in muscle catabolism during lactation (Reese et al., 1984). Thus, to maintain homeostatic levels of creatine for daily cellular energetics as well as continued physical growth and maturation, creatinine levels will be greater because turnover rate is greater. In a separate project, our lab measured creatine, creatine phosphokinase activity, and creatinine levels at post-farrowing and weaning from approximately 250 parity 1 dams (unpublished data). Briefly, our observations indicated there were no differences in creatine between post-farrow or weaning in parity 1 dams. However, plasma creatine phosphokinase activity and plasma levels of creatinine were reduced at weaning in comparison to post-farrowing. Similarly, these parity 1 dams had reduced body condition measurements (body weight, backfat thickness, and loin eye area) at weaning but were on a fixed diet amount (Rempel et al., 2015). The results from these two studies appear juxtaposed to one another, but we believe the differences in plasma creatinine are explainable. Dams in the current study; (1) were approximately 30 days of age younger at farrowing, (2) had approximately 1 pig per litter more, (3) had a 7-day longer lactation period, (4) had a modified genetic basis (use of commercial semen to generate leaner, more industry relevant pigs) and (5) had *ad libitum* access to feed in comparison to our previous study. Likely the females from the current study had greater metabolic demands upon their system requiring added cellular energetics from the creatine pathway.

Other compounds related to energy metabolism were observed within our findings. Phenylacetylglycine, annotated, and hippuric acid, identified, were greater at weaning and both are within the phenylalanine metabolism pathway (Kanehisa et al., 2016). Mice with the functional knock-out for *AHNAK nucleoprotein* gene, a gene associated with calcium homeostasis, are resistant to high-fat diet-induced obesity (Kim et al., 2010). Upon examination of the metabolic perturbation between wild-type and *AHNAK nucleoprotein* knock-out mice fed high-fat or regular chow diets, investigators discovered compounds affecting fat metabolism were altered. Including urinary phenylacetylglycine was elevated in knock-outs on high-fat diets, whilst hippuric acid was only affected by diet. Hippuric acid is also a well-known byproduct of benzene-type aromatic compounds believed to originate from environmental hazards, such as toluene exposure (Beer et al., 1951). Conversely, hippuric acid is also a derivative of dietary protein catabolism (Toromanovic et al., 2008; Pero et al., 2009). Furthermore, evidence suggests that obese subjects have reduced levels of hippuric acid in comparison to lean contemporaries (Salek et al., 2007; Calvani et al., 2010; Won et al., 2013). These data all support the findings in the current study in which first parity dams have elevated levels of phenylacetylglycine and hippuric acid at weaning consistent with some degree (minimal or maximal) of body condition loss over the course of lactation. The increased plasma levels of hippuric acid may be a result of increased dietary protein catabolism and/or mobilized protein from body tissues.

## Conclusion

The metabolic status of first parity dams was altered from early to late lactation regardless of body condition extremes. Interestingly, only two unidentified features were different when evaluating only body condition loss. Future studies are needed to validate the annotated features that did not have a confidence score of 1 and additional techniques to identify the remaining unknown features. Identification of these unknown features may allow for the implementation of strategies to offset tissue immobilization during lactation. However, the current study provides valuable information about the interaction of cellular energetics and reproductive phenotypes. Future investigations will be conducted to determine the dynamic infrastructure between energy and reproduction at the molecular level using plasma metabolomic analyses between first parity dams that fail to return to post-weaning estrus, fail to maintain second pregnancy, or those that successfully rebreed and farrow on their first return cycle.

## Author contributions

LR conceived, designed, and implemented the experiment and prepared the manuscript. JM and WO provided assistance in sample collecting, manuscript preparation and editorial review. CB oversaw the UPLC-MS and GC-MS procedures and provided statistical analyses.

## Disclosure

Mention of trade name, proprietary product, or specified equipment does not constitute a guarantee or warranty by the USDA and does not imply approval to the exclusion of other products that may be suitable. The U.S. Department of Agriculture (USDA) prohibits discrimination in all its programs and activities on the basis of race, color, national origin, age, disability, and where applicable, sex, marital status, familial status, parental status, religion, sexual orientation, genetic information, political beliefs, reprisal, or because all or part of an individual's income is derived from any public assistance program. (Not all prohibited bases apply to all programs.) Persons with disabilities who require alternative means for communication of program information (Braille, large print, audiotape, etc.) should contact USDA's TARGET Center at (202) 720–2600 (voice and TDD). To file a complaint of discrimination, write to USDA, Director, Office of Civil Rights, 1400 Independence Avenue, S.W., Washington, D.C. 20250-9410, or call (800) 795–3272 (voice) or (202) 720–6382 (TDD). USDA is an equal opportunity provider and employer.

### Conflict of interest statement

The authors declare that the research was conducted in the absence of any commercial or financial relationships that could be construed as a potential conflict of interest.
