# Supplementary material for: Non-targeted Plasma Metabolome of Early and Late Lactation Gilts
Source: Front Mol Biosci. 2016 Nov 24;3:77. doi: 10.3389/fmolb.2016.00077 (PMC5122192; doi:10.3389/fmolb.2016.00077)
Supplement: Supplementary file 1 [file Table1.DOCX]

**Supplementary Table 1. Complete list of 770 features detected using GC-MS technique with log_2_ fold-change and ANOVA p-value for PF vs WN and Lo loss vs Hi loss effects.**

| **Annotated compound or Feature identifier^1^** | **PF vs Wn** | | **Lo vs Hi** | |  |  |  |
| --- | --- | --- | --- | --- | --- | --- | --- |
|  | **FC** | **p-value** | **FC** | **p-value** | **Score^2^** | **RT (sec)** | **m/z** |
| C1 | 0 | NS | 0.06 | NS | 4 | 976.26 |  |
| C2 | 0.01 | NS | 0.05 | NS | 4 | 1156.41 |  |
| EDTA | -0.29 | NS | 0.03 | NS | 2 | 810.55 | 580.249 |
| Glucose | -0.02 | NS | -0.04 | NS | 2 | 635.44 | 569 |
| Lactose | -0.13 | NS | -0.02 | NS | 2 | 907.07 | 947 |
| Glucose | -0.01 | NS | 0 | NS | 2 | 627.59 | 569 |
| Cholesterol | -0.01 | NS | -0.01 | NS | 2 | 1026.58 | 458 |
| glucopyranose | 0.05 | NS | 0.25 | NS | 2 | 657.08 | 540 |
| Phenol,2,4-bis(1,1-dimethylethyl)-,phosphite(3:1) | -0.07 | NS | 0.04 | NS | 2 | 1094.2 | 646 |
| Oleic Acid | 1.10 | NS | 0.05 | NS | 2 | 735.79 | 354 |
| Palmitic Acid | 0.14 | NS | -0.01 | NS | 2 | 672.46 | 328 |
| EDTA | 0.04 | NS | -0.32 | 0.1600 | 2 | 789.92 | 580.249 |
| C13 | 0.23 | NS | 0.10 | NS | 4 | 1160.93 |  |
| Phosphate | 0.10 | NS | -0.04 | NS | 2 | 324.9 | 314 |
| Stearic acid | 0.04 | NS | 0.01 | NS | 2 | 744.27 | 356 |
| **Urea** | **-0.44** | **0.0109** | -0.23 | NS | **2** | **302.22** | **204** |
| Alkane | -0.03 | NS | 0.04 | NS | 3 | 1077.44 | 338 |
| C18 | -0.23 | NS | 0.14 | NS | 4 | 803.27 |  |
| Citric acid | 0.07 | NS | -0.06 | NS | 2 | 589.89 | 480 |
| Deoxycholic acid-like | -0.35 | NS | 0.21 | NS | 3 | 1070.42 |  |
| Glycine-3-tms | -0.06 | NS | -0.11 | NS | 2 | 342.57 | 291 |
| Oxalic acid | 0.14 | NS | 0.04 | NS | 2 | 252.29 | 234 |
| Hydrazine | -0.07 | NS | 0.23 | NS | 2 | 754.06 | 320 |
| C24 | -0.01 | NS | 0.02 | NS | 2 | 469.84 |  |
| Lactic Acid | 0.10 | NS | -0.04 | NS | 2 | 210.53 | 234 |
| C26 | 0.03 | NS | 0.02 | NS | 4 | 810.34 |  |
| Alkane | -0.03 | NS | 0.05 | NS | 3 | 981.71 |  |
| **Myo-Inositol** | **0.68** | **0.0224** | -0.02 | NS | **2** | **703.08** | **612** |
| Ornithine | -0.05 | NS | -0.26 | NS | 2 | 588.36 | 420 |
| Alanine | -0.01 | NS | 0.09 | NS | 2 | 232.32 | 233 |
| Glutamine | -0.17 | NS | -0.02 | NS | 2 | 567.51 | 362 |
| Alkane | -0.02 | NS | 0.05 | NS | 3 | 1006.8 |  |
| Alkane | -0.02 | NS | 0.06 | NS | 3 | 1031.87 |  |
| C34 | 0.29 | NS | 0.15 | NS | 4 | 1193.05 |  |
| Alkane | -0.02 | NS | 0.02 | NS | 3 | 1026.59 |  |
| Aminomalonic acid | 0.01 | NS | 0.13 | NS | 2 | 427.33 | 335 |
| C37 | -0.70 | NS | 0.34 | NS | 4 | 1089.29 |  |
| C38 | -0.04 | NS | 0.03 | NS | 4 | 1094.13 |  |
| C39 | 0.35 | NS | 0 | NS | 4 | 315.61 |  |
| C40 | 0.05 | NS | 0.08 | NS | 4 | 1000.24 |  |
| Glutamic acid | 0.06 | NS | 0.01 | NS | 2 | 497.89 | 363 |
| Alkane | -0.02 | NS | 0.04 | NS | 3 | 1048.24 |  |
| C43 | 0.05 | NS | 0.04 | NS | 4 | 810.7 |  |
| Oleamide | -0.33 | NS | -0.23 | NS | 2 | 803.73 | 353 |
| C45 | 0.19 | NS | 0.03 | NS | 4 | 421.37 |  |
| 2-Pyrrolidone carboxylic acid | 0.21 | NS | 0.01 | NS | 2 | 453.18 | 273 |
| **Creatinine** | **-0.66** | **0.0224** | **0.04** | NS | 2 | **471.2** | **329** |
| Carbohydrate | -0.21 | NS | -0.01 | NS | 3 | 370.26 | 496 |
| C49 | 0.23 | NS | 0.11 | NS | 4 | 269.45 |  |
| Carbohydrate | -0.05 | NS | 0.28 | NS | 3 | 889.67 |  |
| **Inositol** | **-1.35** | **0.0016** | **0.10** | NS | 2 | **651.15** | **612** |
| Alkane | -0.02 | NS | 0.04 | NS | 3 | 1014.94 |  |
| C53 | -0.02 | NS | 0.09 | NS | 4 | 1156.39 |  |
| Alkane | -0.02 | NS | 0.04 | NS | 3 | 1110.72 |  |
| Lysine | 0.10 | NS | -0.03 | NS | 2 | 630.23 | 434 |
| Fructose | -0.24 | NS | -0.15 | NS | 2 | 616.37 | 569 |
| Valine | 0.17 | NS | -0.33 | NS | 2 | 292.91 | 261 |
| C58 | -0.11 | NS | -0.01 | NS | 4 | 907.1 |  |
| C59 | 0 | NS | 0 | NS | 4 | 744.4 |  |
| Asparagine[+CO2] | -0.61 | 0.1076 | 0.16 | NS | 2 | 644.19 | 464.201 |
| Threonine | 0.25 | NS | -0.11 | NS | 2 | 384.95 | 335 |
| **Pinitol** | **-1.04** | **0.0024** | 0 | NS | **2** | **599.98** | **554** |
| C63 | 0.01 | NS | 0.23 | NS | 3 | 656.98 |  |
| Isoleucine | -0.04 | NS | -0.23 | NS | 2 | 334.92 | 275.174 |
| Toluic acid | -0.04 | NS | 0.09 | NS | 2 | 930.69 | 208 |
| C66 | -0.02 | NS | -0.26 | NS | 4 | 990.09 |  |
| Carbohydrate | 0.12 | NS | -0.06 | NS | 3 | 677.24 | 540 |
| 1,5-Anhydrohexitol | -0.23 | NS | 0.02 | NS | 2 | 604.88 | 452 |
| C69 | 0.16 | NS | 0.39 | NS | 4 | 623.72 |  |
| C70 | -0.09 | NS | -0.03 | NS | 4 | 635.49 |  |
| Siloxane | 0.30 | NS | -0.03 | NS | 3 | 269.72 |  |
| C72 | -0.08 | NS | 0.01 | NS | 4 | 567.66 |  |
| Phenylalanine | -0.14 | NS | -0.11 | NS | 2 | 503.39 | 309 |
| **Pinitol** | **-1.00** | **0.0024** | 0 | NS | **2** | **600.07** | **554** |
| C75 | 0.01 | NS | -0.04 | NS | 4 | 627.6 |  |
| Glycerol | 0.25 | NS | -0.13 | NS | 2 | 324.58 | 308 |
| Glucopyranose | 0.18 | NS | 0.26 | NS | 2 | 923.71 | 540.261 |
| C78 | 0.02 | NS | 0.06 | NS | 4 | 956.16 |  |
| C79 | -0.04 | NS | 0 | NS | 4 | 1297.7 |  |
| C80 | -0.02 | NS | 0.05 | NS | 4 | 1160.35 |  |
| C81 | -0.33 | NS | -0.08 | NS | 4 | 745.64 |  |
| Pseudouridine | -0.19 | NS | 0.07 | NS | 2 | 786.08 | 604 |
| C83 | -0.03 | NS | -0.16 | NS | 4 | 790.11 |  |
| **Tolyl-glucuronide** | **-1.22** | **5.24E-05** | 0.08 | NS | **2** | **818.02** | **572** |
| Adipic acid | -0.39 | NS | -0.72 | NS | 2 | 541.97 | 377.187 |
| Aspartic acid | -0.03 | NS | 0.23 | NS | 2 | 428.92 | 349 |
| Proline | -0.02 | NS | 0.05 | NS | 2 | 481.29 | 347.122 |
| C88 | -0.13 | NS | 0 | NS | 4 | 1361.94 |  |
| C89 | 0.20 | NS | -0.12 | NS | 4 | 716.97 |  |
| C90 | 0.05 | NS | -0.04 | NS | 4 | 801.36 |  |
| C91 | -0.01 | NS | -0.14 | NS | 4 | 588.54 |  |
| C92 | 0.13 | NS | -0.07 | NS | 4 | 324.85 |  |
| C93 | 0.02 | NS | 0.07 | NS | 4 | 991.39 |  |
| C94 | -0.03 | NS | 0.01 | NS | 4 | 1236.37 |  |
| C95 | 0.01 | NS | 0.07 | NS | 4 | 1195.81 |  |
| C96 | -0.03 | NS | -0.04 | NS | 4 | 1087.08 |  |
| C97 | -0.04 | NS | 0.21 | NS | 4 | 1128.9 |  |
| C98 | -0.16 | NS | 0.27 | NS | 4 | 798.18 |  |
| C99 | 0.04 | NS | 0.07 | NS | 4 | 568.87 |  |
| C100 | 0.09 | NS | -0.01 | NS | 4 | 454.86 |  |
| Lyxonic acid | -0.15 | NS | 0.20 | NS | 2 | 656.45 | 526.245 |
| C102 | 0.07 | NS | 0.30 | NS | 4 | 623.78 |  |
| Disaccharide | 0.18 | NS | 0.22 | NS | 3 | 923.63 | 918 |
| C104 | 0 | NS | 0.02 | NS | 4 | 1313.85 |  |
| C105 | -0.06 | NS | 0.06 | NS | 4 | 1361.42 |  |
| C106 | 0.32 | 0.1992 | -0.11 | NS | 4 | 1299.55 |  |
| C107 | -0.04 | NS | -0.11 | NS | 4 | 1154.84 |  |
| C108 | -0.16 | NS | -0.42 | NS | 4 | 763.55 |  |
| Inositol phosphate | -0.25 | NS | 0.02 | NS | 2 | 819.52 | 764 |
| C110 | 0.28 | NS | 0.18 | NS | 4 | 548.15 |  |
| Asparagine | -0.79 | NS | -0.08 | NS | 2 | 522.77 | 348 |
| C112 | 0.06 | NS | 0.31 | NS | 4 | 528.5 |  |
| Methionine | 0.33 | NS | -0.07 | NS | 2 | 451.1 | 293 |
| M000000_A208005-101-xxx_NA_2073,95_PRED  _VAR5_ALK_NA | 0.16 | NS | 0.24 | NS | 3 | 691.55 |  |
| Gallic acid | -0.12 | NS | 0.01 | NS | 2 | 644.61 | 458 |
| C116 | 0.25 | NS | -0.11 | NS | 4 | 384.97 |  |
| C117 | -0.06 | NS | -0.02 | NS | 4 | 199.53 |  |
| 3-Pyridinol | 0.08 | NS | 0.01 | NS | 2 | 200.38 | 167 |
| Alkane | -0.01 | NS | 0.01 | NS | 3 | 339 |  |
| Glycine | 0.09 | NS | -0.04 | NS | 2 | 240.77 | 219.111 |
| C121 | 0 | NS | 0.04 | NS | 4 | 1394.43 |  |
| C122 | 0.06 | NS | 0.02 | NS | 4 | 1160.84 |  |
| C123 | -0.34 | NS | 0.17 | NS | 4 | 1070.37 |  |
| C124 | 0.11 | NS | -0.03 | NS | 4 | 812.17 |  |
| C125 | 0.02 | NS | 0.01 | NS | 4 | 812.51 |  |
| C126 | -0.04 | NS | -0.08 | NS | 4 | 589.9 |  |
| Proline | 0.07 | NS | -0.29 | NS | 2 | 337.51 | 259.142 |
| C128 | -0.10 | NS | -0.01 | NS | 4 | 907.1 |  |
| C129 | 0.02 | NS | 0.07 | NS | 4 | 981.72 |  |
| C130 | 0 | NS | 0.08 | NS | 4 | 1049.68 |  |
| C131 | 0.03 | NS | 0.11 | NS | 4 | 1039.9 |  |
| Alkane | -0.01 | NS | 0.05 | NS | 3 | 994.3 |  |
| C133 | -0.04 | NS | 0.01 | NS | 4 | 1307.66 |  |
| C134 | -0.04 | NS | 0.07 | NS | 4 | 1225.07 |  |
| Alkane | 0 | NS | 0.07 | NS | 3 | 1057.94 |  |
| 7-Ketocholesterol | -0.01 | NS | 0.02 | NS | 2 | 1055.29 | 472 |
| C137 | 0.01 | NS | 0.08 | NS | 4 | 1118.56 |  |
| C138 | -0.02 | NS | 0.01 | NS | 4 | 1119.81 |  |
| C139 | 0.21 | NS | -0.01 | NS | 4 | 702.99 |  |
| Tryptophan | -0.32 | NS | -0.19 | NS | 2 | 745.29 | 420 |
| 9,12-Octadecadienoic acid | 0.85 | NS | -0.03 | NS | 2 | 734.72 | 352 |
| C142 | -0.02 | NS | -0.03 | NS | 4 | 810.56 |  |
| Myristic acid | 0.13 | NS | -0.02 | NS | 2 | 594.55 | 300 |
| Carbohydrate | 0.15 | NS | -0.19 | NS | 3 | 648.71 |  |
| C145 | -0.38 | NS | -0.01 | NS | 4 | 619.77 |  |
| C146 | -0.31 | NS | -0.03 | NS | 4 | 637.78 |  |
| Mannitol | 1.51 | NS | -0.49 | NS | 2 | 641.02 | 614 |
| Alkane | 0 | NS | 0.05 | NS | 3 | 954.98 |  |
| C149 | -0.15 | NS | -0.10 | NS | 4 | 1026.5 |  |
| C150 | -0.20 | NS | -0.01 | NS | 4 | 1018.44 |  |
| C151 | -0.02 | NS | 0.06 | NS | 4 | 1007.13 |  |
| C152 | -0.06 | NS | 0 | NS | 4 | 1399.79 |  |
| C153 | -0.08 | NS | -0.07 | NS | 4 | 1508.64 |  |
| C154 | 0.02 | NS | 0.04 | NS | 4 | 1244.69 |  |
| C155 | -0.06 | NS | -0.05 | NS | 4 | 1166.17 |  |
| C156 | -0.04 | NS | 0.15 | NS | 4 | 826.07 |  |
| C157 | 0.16 | NS | 0.09 | NS | 4 | 625.56 |  |
| Threonine | 0.27 | NS | -0.11 | NS | 2 | 384.87 | 335 |
| C159 | 0.13 | NS | -0.58 | NS | 4 | 261.82 |  |
| Oxalic acid | 0.57 | NS | -0.11 | NS | 2 | 245.81 | 234 |
| C161 | -0.14 | NS | -0.05 | NS | 4 | 1026.15 |  |
| C162 | 0.05 | NS | 0.07 | NS | 4 | 1325.1 |  |
| C163 | -0.01 | NS | 0 | NS | 4 | 1372.13 |  |
| C164 | 0.03 | NS | 0.09 | NS | 4 | 1251.66 |  |
| C165 | 0.02 | NS | -0.02 | NS | 4 | 1291.42 |  |
| C166 | 0.02 | NS | 0.04 | NS | 4 | 1156.32 |  |
| C167 | -0.04 | NS | 0.07 | NS | 4 | 1072.61 |  |
| C168 | -0.03 | NS | 0.04 | NS | 4 | 1107.88 |  |
| C169 | 0.11 | NS | 0.08 | NS | 4 | 703.38 |  |
| C170 | 0.01 | NS | 0 | NS | 4 | 810.93 |  |
| C171 | 0.42 | NS | -0.27 | NS | 4 | 435.77 |  |
| Lumichrome-like | -0.28 | NS | -0.10 | NS | 3 | 357.92 | 300.133 |
| Toluic acid | 0.05 | NS | 0 | NS | 2 | 373.43 | 208 |
| Leucine | -0.03 | NS | -0.29 | NS | 2 | 322.75 | 275 |
| C175 | -9.45 | NS | 0.05 | NS | 4 | 927.66 |  |
| C176 | 0.05 | NS | 0.10 | NS | 4 | 930.6 |  |
| C177 | -0.07 | NS | 0.03 | NS | 4 | 948.49 |  |
| C178 | -0.01 | NS | 0.05 | NS | 4 | 966.65 |  |
| C179 | -0.03 | NS | 0.10 | NS | 4 | 1421.9 |  |
| C180 | 0.03 | NS | 0.04 | NS | 4 | 1358.9 |  |
| C181 | -0.05 | NS | 0.06 | NS | 4 | 1377.19 |  |
| C182 | -0.02 | NS | 0.07 | NS | 4 | 1505.94 |  |
| C183 | 0.02 | NS | 0.04 | NS | 4 | 1204.62 |  |
| C184 | -0.05 | NS | -0.05 | NS | 4 | 1260.46 |  |
| C185 | 0.35 | NS | 0.24 | NS | 4 | 1293.6 |  |
| C186 | -0.06 | NS | 0.02 | NS | 4 | 1286.95 |  |
| C187 | -0.20 | NS | -0.18 | NS | 4 | 1277.45 |  |
| C188 | 0.08 | NS | 0.05 | NS | 4 | 1160.8 |  |
| C189 | 0.20 | NS | -0.10 | NS | 4 | 1197.03 |  |
| C190 | 0 | NS | 0.03 | NS | 4 | 1066.36 |  |
| C191 | 0.03 | NS | 0.17 | NS | 4 | 1096.27 |  |
| C192 | -0.02 | NS | 0.06 | NS | 4 | 1111.27 |  |
| C193 | 0.02 | NS | -0.04 | NS | 4 | 1143.36 |  |
| C194 | 0 | NS | 0.03 | NS | 4 | 1147.3 |  |
| C195 | 0.22 | NS | 0.05 | NS | 4 | 838.69 |  |
| C196 | 0.12 | NS | -0.34 | NS | 4 | 558.9 |  |
| C197 | -0.22 | NS | 0.11 | NS | 4 | 574.89 |  |
| C198 | 0.01 | NS | -0.01 | NS | 4 | 528.51 |  |
| C199 | 0.29 | NS | 0.03 | NS | 4 | 512.3 |  |
| C200 | -0.07 | NS | -0.01 | NS | 4 | 441.72 |  |
| C201 | -0.01 | NS | 0.02 | NS | 4 | 462.64 |  |
| C202 | 0 | NS | 0 | NS | 4 | 210.93 |  |
| 2-oxo-hexanoic acid | 0.18 | NS | -0.23 | NS | 2 | 290.79 | 231 |
| **C204** | **0.21** | **0.0016** | 0 | NS | **4** | **239.32** |  |
| C205 | -0.03 | NS | 0.19 | NS | 4 | 1041.93 |  |
| C206 | -0.03 | NS | 0.08 | NS | 4 | 1429.02 |  |
| C207 | -0.02 | NS | 0 | NS | 4 | 1425.76 |  |
| C208 | -0.04 | NS | 0.01 | NS | 4 | 1384.21 |  |
| C209 | -0.06 | NS | 0.01 | NS | 4 | 1366.81 |  |
| C210 | 0.03 | NS | 0.08 | NS | 4 | 1368.48 |  |
| C211 | 0 | NS | 0.05 | NS | 4 | 1339.41 |  |
| C212 | -0.02 | NS | 0.02 | NS | 4 | 1465.43 |  |
| C213 | -0.05 | NS | 0.04 | NS | 4 | 1468.19 |  |
| C214 | -0.04 | NS | 0.04 | NS | 4 | 1227.38 |  |
| C215 | 0.01 | NS | 0.05 | NS | 4 | 1213.41 |  |
| C216 | 0.12 | NS | 0 | NS | 4 | 1265.01 |  |
| C217 | -0.05 | NS | 0.13 | NS | 4 | 1275.47 |  |
| C218 | -0.05 | NS | 0.04 | NS | 4 | 1271.49 |  |
| C219 | 0.01 | NS | 0.05 | NS | 4 | 1156.14 |  |
| C220 | -0.02 | NS | 0.11 | NS | 4 | 1188.4 |  |
| C221 | 0.02 | NS | -0.02 | NS | 4 | 1169.91 |  |
| C222 | -0.03 | NS | 0.06 | NS | 4 | 1081.34 |  |
| C223 | -0.07 | NS | 0.04 | NS | 4 | 1061.75 |  |
| C224 | -0.06 | NS | 0.03 | NS | 4 | 1058.5 |  |
| C225 | -0.09 | NS | -0.02 | NS | 4 | 744.42 |  |
| C226 | -0.02 | NS | 0.26 | NS | 4 | 789.45 |  |
| C227 | -0.01 | NS | -0.21 | NS | 4 | 820.07 |  |
| C228 | 0 | NS | -0.01 | NS | 4 | 824.46 |  |
| C229 | -0.66 | NS | -0.08 | NS | 4 | 889.86 |  |
| C230 | 0 | NS | 0.04 | NS | 4 | 860.01 |  |
| C231 | -0.05 | NS | 0.06 | NS | 4 | 506.68 |  |
| C232 | 0.18 | NS | -0.06 | NS | 4 | 493.08 |  |
| C233 | -0.31 | NS | -0.06 | NS | 4 | 465.4 |  |
| C234 | -0.02 | NS | 0.02 | NS | 4 | 627.58 |  |
| C235 | 0.15 | NS | 0.08 | NS | 4 | 930.77 |  |
| C236 | 0.01 | NS | 0.05 | NS | 4 | 975.86 |  |
| C237 | -0.09 | NS | -0.01 | NS | 4 | 1025.94 |  |
| C238 | -0.02 | NS | 0.04 | NS | 4 | 1030.98 |  |
| C239 | 0.07 | NS | 0.20 | NS | 4 | 1038.59 |  |
| C240 | -0.02 | NS | 0.03 | NS | 4 | 1008.78 |  |
| C241 | -0.03 | NS | 0.05 | NS | 4 | 1437.93 |  |
| C242 | -0.02 | NS | 0.11 | NS | 4 | 1440.88 |  |
| C243 | -0.05 | NS | 0 | NS | 4 | 1430.87 |  |
| C244 | 0.01 | NS | -0.05 | NS | 4 | 1382.94 |  |
| C245 | -0.13 | NS | 0.06 | NS | 4 | 1392.98 |  |
| C246 | -0.02 | NS | 0 | NS | 4 | 1320.53 |  |
| C247 | -0.13 | NS | 0.13 | NS | 4 | 1375.03 |  |
| C248 | 0.10 | NS | -0.06 | NS | 4 | 1509.9 |  |
| C249 | -0.28 | NS | 0.11 | NS | 4 | 1206.08 |  |
| C250 | -0.13 | NS | 0.10 | NS | 4 | 1211.67 |  |
| C251 | -0.04 | NS | 0.06 | NS | 4 | 1243.96 |  |
| C252 | -0.02 | NS | 0.05 | NS | 4 | 1256.27 |  |
| C253 | 0.01 | NS | -0.06 | NS | 4 | 1294.37 |  |
| C254 | -0.07 | NS | 0 | NS | 4 | 1285.63 |  |
| C255 | -0.08 | NS | 0.02 | NS | 4 | 1288.07 |  |
| C256 | 0 | NS | 0.04 | NS | 4 | 1275.22 |  |
| C257 | 0.02 | NS | 0.04 | NS | 4 | 1181.86 |  |
| C258 | 0.07 | NS | -0.05 | NS | 4 | 1171.72 |  |
| C259 | -0.05 | NS | 0.01 | NS | 4 | 1057.45 |  |
| C260 | 0 | NS | 0.05 | NS | 4 | 1086.6 |  |
| C261 | -0.07 | NS | 0.09 | NS | 4 | 1097.34 |  |
| **C262** | **-0.49** | **0.0039** | 0.08 | NS | **4** | **1107.14** |  |
| C263 | -0.05 | NS | 0.04 | NS | 4 | 1112.96 |  |
| C264 | -0.14 | NS | 0.14 | NS | 4 | 1110.3 |  |
| C265 | 0.02 | NS | 0 | NS | 4 | 1124.73 |  |
| C266 | -0.03 | NS | -0.03 | NS | 4 | 744.45 |  |
| C267 | 0.04 | NS | 0.04 | NS | 4 | 810.88 |  |
| Sorbose | -0.30 | NS | -0.12 | NS | 2 | 523.01 | 569 |
| **C269** | **-0.28** | **0.0109** | 0.08 | NS | **4** | **675.78** |  |
| C270 | -0.09 | NS | 0 | NS | 4 | 604.63 |  |
| C271 | 0.03 | NS | 0.05 | NS | 4 | 630.08 |  |
| C272 | -0.15 | NS | -0.10 | NS | 4 | 617.18 |  |
| Pyruvic acid | 0.26 | NS | -0.02 | NS | 2 | 205.29 | 189.082 |
| n-Butylamine | 0.17 | 0.1612 | 0.06 | NS | 2 | 292.59 | 217 |
| C275 | 0 | NS | 0.03 | NS | 4 | 343.7 |  |
| C276 | -0.10 | NS | 0 | NS | 4 | 907.56 |  |
| C277 | -0.44 | NS | -0.05 | NS | 4 | 947.23 |  |
| C278 | -0.01 | NS | 0.05 | NS | 4 | 941.43 |  |
| C279 | -0.02 | NS | 0.04 | NS | 4 | 954.58 |  |
| C280 | 0 | NS | 0.04 | NS | 4 | 984.95 |  |
| C281 | -0.04 | NS | 0.05 | NS | 4 | 1024.45 |  |
| C282 | -0.02 | NS | 0.05 | NS | 4 | 1033.6 |  |
| C283 | -0.27 | NS | -0.23 | NS | 4 | 1037.5 |  |
| C284 | 0.04 | NS | 0.01 | NS | 4 | 1015.14 |  |
| C285 | -0.02 | NS | 0.02 | NS | 4 | 1003.02 |  |
| C286 | 0 | NS | 0.05 | NS | 4 | 999.35 |  |
| C287 | -0.13 | NS | 0.01 | NS | 4 | 1452.43 |  |
| C288 | 0 | NS | 0.04 | NS | 4 | 1434.27 |  |
| C289 | -0.05 | NS | -0.04 | NS | 4 | 1447.91 |  |
| C290 | -0.10 | NS | 0 | NS | 4 | 1416.42 |  |
| C291 | -0.17 | NS | -0.06 | NS | 4 | 1413.71 |  |
| C292 | 0.01 | NS | -0.01 | NS | 4 | 1391.52 |  |
| C293 | 0.03 | NS | 0.13 | NS | 4 | 1401.65 |  |
| C294 | -0.05 | NS | 0.04 | NS | 4 | 1315.42 |  |
| C295 | 0 | NS | 0.01 | NS | 4 | 1325.37 |  |
| C296 | 0.01 | NS | 0.12 | NS | 4 | 1357.84 |  |
| C297 | -0.01 | NS | 0.04 | NS | 4 | 1352.44 |  |
| C298 | 0.03 | NS | -0.01 | NS | 4 | 1354.15 |  |
| C299 | 0 | NS | 0.05 | NS | 4 | 1346.95 |  |
| C300 | 0.03 | NS | 0.04 | NS | 4 | 1334.12 |  |
| C301 | -0.04 | NS | 0.02 | NS | 4 | 1490.6 |  |
| C302 | 0.02 | NS | 0.08 | NS | 4 | 1497.68 |  |
| C303 | -0.05 | NS | 0.21 | NS | 4 | 1208.63 |  |
| C304 | -0.02 | NS | 0.06 | NS | 4 | 1214 |  |
| C305 | 0 | NS | 0.01 | NS | 4 | 1219.04 |  |
| C306 | -0.09 | NS | -0.03 | NS | 4 | 1264.97 |  |
| C307 | 0 | NS | 0.10 | NS | 4 | 1257.4 |  |
| C308 | -0.09 | NS | 0.01 | NS | 4 | 1253.53 |  |
| C309 | 0.02 | NS | 0.09 | NS | 4 | 1279.73 |  |
| C310 | -0.20 | NS | 0.04 | NS | 4 | 1186.21 |  |
| C311 | -0.01 | NS | 0.08 | NS | 4 | 1177.39 |  |
| C312 | -0.06 | NS | 0.01 | NS | 4 | 1077.87 |  |
| C313 | -0.52 | NS | 0.23 | NS | 4 | 1069.86 |  |
| C314 | -0.17 | NS | 0.07 | NS | 4 | 1064.72 |  |
| C315 | -0.05 | NS | -0.01 | NS | 4 | 1063.3 |  |
| C316 | 0.01 | NS | 0.07 | NS | 4 | 1138.48 |  |
| C317 | 0 | NS | 0 | NS | 4 | 1136.43 |  |
| C318 | -0.06 | NS | -0.18 | NS | 4 | 769.03 |  |
| C319 | 0.05 | NS | -0.52 | NS | 4 | 758.11 |  |
| C320 | -0.24 | NS | 0.34 | NS | 4 | 761.79 |  |
| Heptadecanoic acid | 0.01 | NS | -0.02 | NS | 2 | 709.14 | 342.295 |
| C322 | -0.12 | NS | 0.34 | NS | 4 | 728.21 |  |
| C323 | 0.50 | NS | -0.21 | NS | 4 | 812.28 |  |
| C324 | 0.12 | NS | -0.17 | NS | 4 | 777.42 |  |
| C325 | -0.05 | NS | -0.11 | NS | 4 | 793.26 |  |
| C326 | -0.24 | NS | -0.36 | NS | 4 | 477.12 |  |
| C327 | -0.01 | NS | -0.01 | NS | 4 | 635.41 |  |
| **C328** | **-0.75** | **4.61E-14** | -0.01 | NS | **4** | **348.45** |  |
| C329 | 0.01 | NS | 0.03 | NS | 4 | 345.79 |  |
| C330 | 0 | NS | 0.04 | NS | 4 | 330.3 |  |
| C331 | 0.09 | NS | 0.14 | NS | 4 | 322.33 |  |
| C332 | 0.04 | NS | 0.06 | NS | 4 | 322.3 |  |
| C333 | 0.06 | NS | 0.04 | NS | 4 | 256.65 |  |
| C334 | -0.32 | NS | -0.13 | NS | 4 | 909.73 |  |
| C335 | 0 | NS | 0.02 | NS | 4 | 977.44 |  |
| C336 | -0.09 | NS | 0.12 | NS | 4 | 1047.34 |  |
| C337 | -0.16 | NS | -0.14 | NS | 4 | 1035.23 |  |
| C338 | 0 | NS | 0.08 | NS | 4 | 1035.04 |  |
| C339 | -0.02 | NS | -0.08 | NS | 4 | 989.23 |  |
| C340 | -0.12 | NS | 0.06 | NS | 4 | 1454.85 |  |
| C341 | -0.12 | NS | 0.01 | NS | 4 | 1456.32 |  |
| C342 | -0.01 | NS | -0.01 | NS | 4 | 1415.37 |  |
| C343 | 0.03 | NS | 0 | NS | 4 | 1408.13 |  |
| C344 | 0.01 | NS | 0.10 | NS | 4 | 1419.45 |  |
| C345 | -0.02 | NS | 0.06 | NS | 4 | 1397.96 |  |
| C346 | -0.02 | NS | 0.03 | NS | 4 | 1309.41 |  |
| C347 | -0.07 | NS | 0.02 | NS | 4 | 1310.66 |  |
| C348 | -0.04 | NS | -0.03 | NS | 4 | 1328.49 |  |
| C349 | -0.10 | NS | -0.04 | NS | 4 | 1318.95 |  |
| C350 | -0.01 | NS | 0 | NS | 4 | 1322.81 |  |
| C351 | 0.08 | NS | 0.11 | NS | 4 | 1356.56 |  |
| C352 | -0.02 | NS | 0.04 | NS | 4 | 1365.38 |  |
| C353 | 0 | NS | 0 | NS | 4 | 1370.31 |  |
| C354 | -0.08 | NS | -0.02 | NS | 4 | 1349.54 |  |
| C355 | -0.02 | NS | -0.02 | NS | 4 | 1341.16 |  |
| C356 | -0.03 | NS | 0.09 | NS | 4 | 1335.63 |  |
| C357 | -0.14 | NS | -0.01 | NS | 4 | 1331.87 |  |
| C358 | 0.10 | NS | 0 | NS | 4 | 1463.54 |  |
| C359 | -0.08 | NS | 0.02 | NS | 4 | 1481.07 |  |
| C360 | -0.01 | NS | -0.05 | NS | 4 | 1226.02 |  |
| C361 | 0.06 | NS | 0.04 | NS | 4 | 1238.3 |  |
| C362 | -0.04 | NS | 0 | NS | 4 | 1230.4 |  |
| C363 | -0.04 | NS | 0.01 | NS | 4 | 1231.04 |  |
| C364 | 0.05 | NS | 0.05 | NS | 4 | 1207.57 |  |
| C365 | -0.09 | NS | 0.04 | NS | 4 | 1204.08 |  |
| C366 | -0.12 | NS | 0.07 | NS | 4 | 1214.84 |  |
| C367 | 0.22 | NS | 0.04 | NS | 4 | 1216.51 |  |
| C368 | -0.04 | NS | 0 | NS | 4 | 1263.35 |  |
| C369 | -0.11 | NS | 0.07 | NS | 4 | 1249.59 |  |
| C370 | 0 | NS | -0.06 | NS | 4 | 1247.67 |  |
| C371 | -0.08 | NS | -0.07 | NS | 4 | 1295.81 |  |
| C372 | -0.03 | NS | 0.03 | NS | 4 | 1301.29 |  |
| C373 | -0.11 | NS | -0.10 | NS | 4 | 1282.82 |  |
| C374 | 0.05 | NS | 0.06 | NS | 4 | 1280.83 |  |
| C375 | 0.01 | NS | 0.09 | NS | 4 | 1158.6 |  |
| C376 | -0.09 | NS | 0 | NS | 4 | 1182.68 |  |
| C377 | -0.06 | NS | 0.08 | NS | 4 | 1181.28 |  |
| C378 | -0.03 | NS | -0.08 | NS | 4 | 1179.47 |  |
| C379 | 0.03 | NS | 0.02 | NS | 4 | 1174.32 |  |
| C380 | -0.04 | NS | -0.02 | NS | 4 | 1091.33 |  |
| C381 | -0.01 | NS | 0.11 | NS | 4 | 1108.43 |  |
| C382 | -0.06 | NS | 0.07 | NS | 4 | 1105.41 |  |
| C383 | -0.01 | NS | -0.11 | NS | 4 | 1135.61 |  |
| C384 | 0.21 | NS | -0.33 | NS | 4 | 704.53 |  |
| C385 | -0.05 | NS | 0 | NS | 4 | 812.48 |  |
| C386 | 0.80 | NS | 0.03 | NS | 4 | 803.96 |  |
| Pyridoxamine | -0.19 | NS | -0.08 | NS | 2 | 775.28 | 384.208 |
| C388 | -0.17 | NS | -0.08 | NS | 4 | 786.03 |  |
| C389 | 0.33 | NS | -0.20 | NS | 4 | 783.86 |  |
| C390 | 0.14 | NS | 0.18 | NS | 4 | 831.24 |  |
| C391 | -0.06 | NS | 0.03 | NS | 4 | 895.69 |  |
| C392 | 0.19 | NS | -0.01 | NS | 4 | 863.36 |  |
| C393 | 0.45 | NS | -0.07 | NS | 4 | 544.43 |  |
| C394 | -0.33 | NS | -0.03 | NS | 4 | 576.69 |  |
| **C395** | **-0.94** | **0.0411** | -0.12 | NS | **4** | **565.37** |  |
| C396 | 0.08 | NS | 0.20 | NS | 4 | 433.41 |  |
| Hydroxyproline | 0.11 | NS | 0.03 | NS | 2 | 454.84 | 347 |
| C398 | -0.48 | NS | -0.42 | NS | 4 | 476.85 |  |
| C399 | 0.11 | NS | -0.07 | NS | 4 | 473.35 |  |
| C400 | 0.09 | NS | -0.05 | NS | 4 | 677.21 |  |
| C401 | -0.20 | NS | -0.08 | NS | 4 | 668.19 |  |
| C402 | -0.01 | NS | 0.02 | NS | 4 | 627.45 |  |
| C403 | -0.16 | NS | 0.01 | NS | 4 | 637.82 |  |
| Alanine | 0.13 | NS | 0.09 | NS | 2 | 370.57 | 305.166 |
| C405 | 0.04 | NS | 0.04 | NS | 4 | 229.54 |  |
| 4C406 | -0.02 | NS | 0.01 | NS | 4 | 222.68 |  |
| **C407** | **0.28** | **0.0003** | -0.05 | NS | **4** | **302.49** |  |
| C408 | 0.40 | NS | -0.24 | NS | 4 | 322.92 |  |
| C409 | 0.10 | NS | -0.03 | NS | 4 | 269.46 |  |
| C410 | -0.13 | NS | -0.04 | NS | 4 | 946.42 |  |
| C411 | -0.04 | NS | 0.03 | NS | 4 | 966.53 |  |
| C412 | -0.01 | NS | 0.02 | NS | 4 | 1028.89 |  |
| C413 | 0 | NS | 0.01 | NS | 4 | 1436.2 |  |
| C414 | -0.05 | NS | -0.05 | NS | 4 | 1442.22 |  |
| C415 | -0.05 | NS | -0.03 | NS | 4 | 1449.09 |  |
| C416 | -0.15 | NS | 0.02 | NS | 4 | 1446.21 |  |
| C417 | 0.02 | NS | 0.02 | NS | 4 | 1410.7 |  |
| C418 | 0 | NS | -0.03 | NS | 4 | 1423.52 |  |
| C419 | -0.02 | NS | 0.02 | NS | 4 | 1385.96 |  |
| C420 | 0 | NS | 0.10 | NS | 4 | 1396.47 |  |
| C421 | -0.01 | NS | 0.06 | NS | 4 | 1404.89 |  |
| C422 | 0 | NS | 0.12 | NS | 4 | 1403.16 |  |
| C423 | -0.05 | NS | 0.09 | NS | 4 | 1312.2 |  |
| C424 | 0 | NS | -0.08 | NS | 4 | 1305.7 |  |
| C425 | 0.09 | NS | 0.04 | NS | 4 | 1304.49 |  |
| C426 | -0.04 | NS | 0.05 | NS | 4 | 1363.64 |  |
| C427 | 0.13 | NS | 0.20 | NS | 4 | 1379.17 |  |
| C428 | -0.02 | NS | 0.02 | NS | 4 | 1351.64 |  |
| C429 | -0.01 | NS | -0.06 | NS | 4 | 1354.16 |  |
| C430 | -0.01 | NS | 0.05 | NS | 4 | 1342.25 |  |
| C431 | 0 | NS | 0.08 | NS | 4 | 1337.34 |  |
| C432 | -0.01 | NS | 0.09 | NS | 4 | 1509.76 |  |
| C433 | -0.07 | NS | -0.14 | NS | 4 | 1496.19 |  |
| C434 | -0.06 | NS | 0.06 | NS | 4 | 1466.32 |  |
| C435 | -0.09 | NS | 0.12 | NS | 4 | 1470.22 |  |
| C436 | 0.03 | NS | -0.07 | NS | 4 | 1476.63 |  |
| C437 | -0.03 | NS | -0.02 | NS | 4 | 1478.84 |  |
| C438 | -0.03 | NS | 0 | NS | 4 | 1486.96 |  |
| C439 | -0.07 | NS | 0.07 | NS | 4 | 1221.34 |  |
| C440 | 0.05 | NS | 0.03 | NS | 4 | 1222.59 |  |
| C441 | -0.03 | NS | 0.06 | NS | 4 | 1228.76 |  |
| C442 | 0 | NS | 0.01 | NS | 4 | 1227 |  |
| C443 | -0.04 | NS | 0.04 | NS | 4 | 1233.23 |  |
| C444 | -0.24 | NS | -0.13 | NS | 4 | 1234.51 |  |
| C445 | -0.05 | NS | 0.12 | NS | 4 | 1220.09 |  |
| C446 | 0 | NS | 0.02 | NS | 4 | 1262.56 |  |
| C447 | -0.01 | NS | 0.05 | NS | 4 | 1241.49 |  |
| C448 | 0.03 | NS | -0.05 | NS | 4 | 1240.43 |  |
| C449 | 0.04 | NS | -0.02 | NS | 4 | 1246.33 |  |
| C450 | -0.08 | NS | 0.03 | NS | 4 | 1243.97 |  |
| C451 | -0.10 | NS | 0.02 | NS | 4 | 1248.5 |  |
| C452 | 0.03 | NS | -0.11 | NS | 4 | 1302.23 |  |
| C453 | -0.02 | NS | -0.03 | NS | 4 | 1289.87 |  |
| C454 | -0.02 | NS | -0.01 | NS | 4 | 1284 |  |
| C455 | 0.02 | NS | 0.10 | NS | 4 | 1269.42 |  |
| C456 | -0.09 | NS | 0.01 | NS | 4 | 1270.14 |  |
| C457 | 0.16 | NS | -0.08 | NS | 4 | 1267.92 |  |
| C458 | 0.26 | 0.1481 | -0.04 | NS | 4 | 1153.63 |  |
| C459 | 0.02 | NS | 0.10 | NS | 4 | 1183.39 |  |
| C460 | 0.03 | NS | 0.04 | NS | 4 | 1184.6 |  |
| C461 | 0.05 | NS | -0.05 | NS | 4 | 1178.19 |  |
| C462 | -0.02 | NS | -0.05 | NS | 4 | 1175.82 |  |
| C463 | 0.06 | NS | 0.16 | NS | 4 | 1173.35 |  |
| C464 | -0.03 | NS | 0.09 | NS | 4 | 1168.31 |  |
| C465 | 0 | NS | -0.06 | NS | 4 | 1075.84 |  |
| C466 | -0.22 | NS | 0.01 | NS | 4 | 1060.88 |  |
| C467 | -0.23 | NS | 0.18 | NS | 4 | 1053.56 |  |
| C468 | 0.11 | NS | 0 | NS | 4 | 1121.68 |  |
| C469 | -0.04 | NS | 0.23 | NS | 4 | 1114.22 |  |
| C470 | -0.07 | NS | 0.07 | NS | 4 | 1141.29 |  |
| C471 | 0.04 | NS | 0.15 | NS | 4 | 1149.25 |  |
| C472 | 0.03 | NS | -0.11 | NS | 4 | 1138.22 |  |
| C473 | 0.04 | NS | -0.04 | NS | 4 | 1133.49 |  |
| C474 | -0.08 | NS | 0 | NS | 4 | 1130.52 |  |
| C475 | -0.22 | NS | -0.13 | NS | 4 | 731.33 |  |
| C476 | -0.09 | NS | -0.02 | NS | 4 | 796.39 |  |
| C477 | -0.16 | NS | -0.10 | NS | 4 | 801.57 |  |
| C478 | -0.17 | NS | -0.50 | NS | 4 | 789.27 |  |
| C479 | -0.13 | NS | -0.06 | NS | 4 | 822.63 |  |
| C480 | 0.21 | NS | -0.14 | NS | 4 | 837.92 |  |
| C481 | 0.03 | NS | 0.07 | NS | 4 | 877.11 |  |
| **Psicose** | **-0.68** | **0.0731** | 0.16 | NS | **2** | **549.71** | **569.288** |
| C483 | -0.01 | NS | 0.04 | NS | 4 | 561.19 |  |
| C484 | -0.35 | NS | -0.14 | NS | 4 | 522.89 |  |
| C485 | 0.07 | NS | -0.03 | NS | 4 | 670.68 |  |
| C486 | -0.01 | NS | 0.22 | NS | 4 | 601.7 |  |
| C487 | 0.47 | NS | 0.02 | NS | 4 | 589.91 |  |
| **C488** | **1.83** | **0.0148** | -0.15 | NS | **4** | **621.97** |  |
| C489 | 0.06 | NS | -0.01 | NS | 4 | 630.29 |  |
| C490 | 0.13 | NS | 0.05 | NS | 4 | 370.56 |  |
| C491 | 0.10 | NS | -0.01 | NS | 4 | 368.76 |  |
| C492 | 0.16 | NS | -0.35 | NS | 4 | 226.33 |  |
| C493 | 0.07 | NS | 0.02 | NS | 4 | 252.44 |  |
| C494 | -0.15 | NS | -0.04 | NS | 4 | 912.14 |  |
| C495 | -0.01 | NS | 0.06 | NS | 4 | 920.31 |  |
| C496 | 0.01 | NS | -0.16 | NS | 4 | 958.96 |  |
| C497 | -0.02 | NS | 0.06 | NS | 4 | 955.32 |  |
| C498 | -0.01 | NS | 0.05 | NS | 4 | 973.52 |  |
| C499 | 0.01 | NS | -0.04 | NS | 4 | 1032.55 |  |
| C500 | -0.02 | NS | 0.08 | NS | 4 | 993.54 |  |
| C501 | -0.02 | NS | 0.03 | NS | 4 | 988.71 |  |
| C502 | 0 | NS | 0.05 | NS | 4 | 991.35 |  |
| C503 | -0.09 | NS | 0.01 | NS | 4 | 1014.2 |  |
| C504 | -0.12 | NS | 0.11 | NS | 4 | 1010.31 |  |
| C505 | 0 | NS | 0.13 | NS | 4 | 1453.68 |  |
| C506 | 0.16 | NS | -0.01 | NS | 4 | 1458.08 |  |
| C507 | 0 | NS | 0.01 | NS | 4 | 1443.52 |  |
| C508 | -0.05 | NS | 0 | NS | 4 | 1445.17 |  |
| C509 | 0.09 | NS | -0.05 | NS | 4 | 1427.78 |  |
| C510 | -0.04 | NS | -0.01 | NS | 4 | 1418.03 |  |
| C511 | -0.12 | NS | 0.18 | NS | 4 | 1388.04 |  |
| C512 | -0.03 | NS | 0.21 | NS | 4 | 1381.78 |  |
| C513 | -0.03 | NS | -0.08 | NS | 4 | 1389.92 |  |
| C514 | 0.05 | NS | -0.05 | NS | 4 | 1390.28 |  |
| C515 | -0.03 | NS | 0.08 | NS | 4 | 1394.92 |  |
| C516 | -0.23 | NS | -0.24 | NS | 4 | 1405.7 |  |
| C517 | -0.10 | NS | -0.03 | NS | 4 | 1404.7 |  |
| C518 | -0.07 | NS | -0.02 | NS | 4 | 1314.2 |  |
| C519 | -0.11 | NS | -0.05 | NS | 4 | 1327.36 |  |
| C520 | -0.07 | NS | -0.02 | NS | 4 | 1321.4 |  |
| C521 | 0.02 | NS | 0.12 | NS | 4 | 1343.48 |  |
| C522 | -0.08 | NS | -0.02 | NS | 4 | 1344.55 |  |
| C523 | -0.07 | NS | 0.01 | NS | 4 | 1347.79 |  |
| C524 | -0.07 | NS | 0.05 | NS | 4 | 1348.95 |  |
| C525 | -0.04 | NS | 0.06 | NS | 4 | 1337 |  |
| C526 | 0.07 | NS | 0.03 | NS | 4 | 1333.06 |  |
| C527 | -0.17 | NS | 0.05 | NS | 4 | 1333.81 |  |
| C528 | 0.02 | NS | -0.12 | NS | 4 | 1507.59 |  |
| C529 | -0.14 | NS | -0.04 | NS | 4 | 1492.25 |  |
| C530 | -0.09 | NS | -0.05 | NS | 4 | 1495.15 |  |
| C531 | 0 | NS | -0.04 | NS | 4 | 1499.36 |  |
| C532 | -0.07 | NS | 0.04 | NS | 4 | 1473.1 |  |
| C533 | -0.04 | NS | 0.04 | NS | 4 | 1475.33 |  |
| C534 | 0.07 | NS | 0.06 | NS | 4 | 1482.85 |  |
| C535 | 0.05 | NS | 0.07 | NS | 4 | 1223.64 |  |
| C536 | -0.02 | NS | -0.04 | NS | 4 | 1223.44 |  |
| C537 | -0.04 | NS | 0.06 | NS | 4 | 1202.42 |  |
| C538 | -0.04 | NS | 0.05 | NS | 4 | 1258.81 |  |
| C539 | -0.02 | NS | 0.04 | NS | 4 | 1266.38 |  |
| C540 | 0 | NS | 0.02 | NS | 4 | 1243.19 |  |
| C541 | -0.10 | NS | 0.13 | NS | 4 | 1255.25 |  |
| C542 | 0.02 | NS | -0.01 | NS | 4 | 1288.83 |  |
| C543 | 0.16 | NS | -0.05 | NS | 4 | 1164.08 |  |
| C544 | 0.03 | NS | 0.08 | NS | 4 | 1159.27 |  |
| C545 | -0.11 | NS | 0.03 | NS | 4 | 1159.12 |  |
| C546 | 0.03 | NS | 0.05 | NS | 4 | 1191.69 |  |
| C547 | 0.08 | NS | 0.08 | NS | 4 | 1190.93 |  |
| C548 | 0 | NS | -0.10 | NS | 4 | 1195.19 |  |
| C549 | -0.06 | NS | 0.02 | NS | 4 | 1198.45 |  |
| C550 | -0.11 | NS | 0.02 | NS | 4 | 1200.41 |  |
| C551 | -0.03 | NS | 0.16 | NS | 4 | 1079.57 |  |
| C552 | -0.01 | NS | -0.02 | NS | 4 | 1099.23 |  |
| C553 | -0.10 | NS | 0 | NS | 4 | 1098.1 |  |
| C554 | -0.15 | NS | -0.07 | NS | 4 | 1117.12 |  |
| C555 | -0.07 | NS | 0 | NS | 4 | 1102.19 |  |
| C556 | 0.32 | NS | 0.16 | NS | 4 | 1101.36 |  |
| C557 | 0.03 | NS | 0.04 | NS | 4 | 1107.46 |  |
| C558 | 0 | NS | -0.01 | NS | 4 | 1131.44 |  |
| C559 | -0.13 | NS | 0.05 | NS | 4 | 1127.87 |  |
| C560 | 0.71 | NS | -0.06 | NS | 4 | 770.67 |  |
| C561 | 0.25 | NS | 0.02 | NS | 4 | 747.35 |  |
| C562 | 0.02 | NS | 0 | NS | 4 | 711.38 |  |
| C563 | -0.04 | NS | 0.10 | NS | 4 | 739.86 |  |
| C564 | 0.06 | NS | -0.02 | NS | 4 | 725.63 |  |
| C565 | 0.12 | NS | -0.03 | NS | 4 | 780.73 |  |
| C566 | 0.49 | NS | -0.10 | NS | 4 | 832.73 |  |
| C567 | 0.15 | NS | -0.11 | NS | 4 | 845.77 |  |
| C568 | -0.14 | NS | 0.03 | NS | 4 | 844.1 |  |
| C569 | -0.04 | NS | 0 | NS | 4 | 866.38 |  |
| C570 | 0.10 | NS | -0.03 | NS | 4 | 421.77 |  |
| **C571** | **-0.14** | **0.0726** | 0.02 | NS | **4** | **469.96** |  |
| **C572** | **-0.15** | **0.0224** | -0.02 | NS | **4** | **594.62** |  |
| C573 | -0.06 | NS | 0.21 | NS | 4 | 652.72 |  |
| C574 | -0.01 | NS | 0.22 | NS | 4 | 652.7 |  |
| **C575** | **-1.10** | **2.62E-05** | 0.18 | NS | **4** | **650.06** |  |
| C576 | 0.10 | NS | 0.09 | NS | 4 | 627.6 |  |
| C577 | 0.07 | NS | 0.01 | NS | 4 | 381.29 |  |
| C578 | 0.13 | NS | 0 | NS | 4 | 308.7 |  |
| C579 | 0.09 | NS | -0.14 | NS | 4 | 292.79 |  |
| C580 | 0.11 | NS | -0.10 | NS | 4 | 292.72 |  |
| C581 | -0.13 | NS | -0.18 | NS | 4 | 337.51 |  |
| **C582** | **0.23** | **0.0817** | 0.12 | NS | **4** | **247.66** |  |
| C583 | 0.16 | NS | -0.09 | NS | 4 | 242.59 |  |
| C584 | 0.04 | NS | -0.01 | NS | 4 | 936.97 |  |
| **C585** | **-0.28** | **0.0388** | -0.01 | NS | **4** | **960.51** |  |
| C586 | -0.04 | NS | 0 | NS | 4 | 961.91 |  |
| C587 | -0.29 | NS | 0.16 | NS | 4 | 954.23 |  |
| C588 | 0 | NS | 0.04 | NS | 4 | 955.76 |  |
| C589 | -0.02 | NS | -0.02 | NS | 4 | 980.24 |  |
| C590 | 0.05 | NS | -0.06 | NS | 4 | 1044.18 |  |
| C591 | -0.08 | NS | 0.09 | NS | 4 | 1023.47 |  |
| C592 | -0.01 | NS | 0 | NS | 4 | 992.62 |  |
| C593 | 0.01 | NS | 0.05 | NS | 4 | 1012.57 |  |
| C594 | 0.01 | NS | 0.03 | NS | 4 | 1021.91 |  |
| C595 | -0.12 | NS | -0.08 | NS | 4 | 1458.17 |  |
| C596 | -0.10 | NS | -0.01 | NS | 4 | 1438.93 |  |
| C597 | -0.24 | NS | 0.06 | NS | 4 | 1449.93 |  |
| C598 | -0.07 | NS | 0.06 | NS | 4 | 1444.31 |  |
| C599 | -0.02 | NS | -0.03 | NS | 4 | 1414.65 |  |
| C600 | 0.13 | NS | 0.17 | NS | 4 | 1412.3 |  |
| C601 | -0.22 | NS | -0.02 | NS | 4 | 1410.86 |  |
| C602 | 0.01 | NS | 0 | NS | 4 | 1388.35 |  |
| C603 | 0.02 | NS | 0.06 | NS | 4 | 1386.73 |  |
| C604 | 0.09 | NS | 0.11 | NS | 4 | 1380.88 |  |
| C605 | 0.08 | NS | 0.16 | NS | 4 | 1402.8 |  |
| C606 | 0.15 | NS | 0.13 | NS | 4 | 1309 |  |
| C607 | 0.09 | NS | 0.11 | NS | 4 | 1310.8 |  |
| C608 | 0.05 | NS | 0.06 | NS | 4 | 1326.57 |  |
| C609 | 0.04 | NS | 0.04 | NS | 4 | 1317.23 |  |
| C610 | -0.16 | NS | -0.02 | NS | 4 | 1316.78 |  |
| C611 | -0.03 | NS | -0.10 | NS | 4 | 1377.55 |  |
| C612 | -0.03 | NS | -0.01 | NS | 4 | 1373.4 |  |
| C613 | 0 | NS | 0.05 | NS | 4 | 1347.07 |  |
| C614 | 0 | NS | 0.12 | NS | 4 | 1504.43 |  |
| C615 | -0.05 | NS | -0.10 | NS | 4 | 1509.38 |  |
| C616 | 0.02 | NS | 0.09 | NS | 4 | 1464 |  |
| C617 | -0.07 | NS | -0.06 | NS | 4 | 1471.7 |  |
| C618 | 0.13 | NS | 0 | NS | 4 | 1485.36 |  |
| C619 | -0.08 | NS | 0.01 | NS | 4 | 1484.24 |  |
| C620 | 0.03 | NS | -0.05 | NS | 4 | 1235.47 |  |
| C621 | -0.06 | NS | 0.09 | NS | 4 | 1232.5 |  |
| C622 | 0 | NS | 0.11 | NS | 4 | 1234.33 |  |
| C623 | -0.03 | NS | 0.14 | NS | 4 | 1210.02 |  |
| C624 | 0 | NS | 0.05 | NS | 4 | 1217.92 |  |
| C625 | -0.06 | NS | 0.13 | NS | 4 | 1262.35 |  |
| C626 | 0.02 | NS | -0.03 | NS | 4 | 1253.77 |  |
| C627 | 0.01 | NS | 0.11 | NS | 4 | 1255.01 |  |
| C628 | -0.11 | NS | 0.02 | NS | 4 | 1254.54 |  |
| C629 | -0.04 | NS | -0.08 | NS | 4 | 1293.19 |  |
| C630 | -0.01 | NS | 0.15 | NS | 4 | 1274.61 |  |
| C631 | -0.05 | NS | 0.06 | NS | 4 | 1278.56 |  |
| C632 | -0.12 | NS | -0.02 | NS | 4 | 1272.88 |  |
| C633 | 0.06 | NS | 0.03 | NS | 4 | 1162.58 |  |
| C634 | 0.01 | NS | 0.01 | NS | 4 | 1161.44 |  |
| C635 | -0.06 | NS | 0.02 | NS | 4 | 1193.47 |  |
| C636 | -0.13 | NS | 0.04 | NS | 4 | 1199.5 |  |
| C637 | -0.09 | NS | -0.04 | NS | 4 | 1180.85 |  |
| C638 | 0 | NS | 0.21 | NS | 4 | 1068.46 |  |
| C639 | -0.11 | NS | -0.02 | NS | 4 | 1068.07 |  |
| C640 | -0.06 | NS | 0.15 | NS | 4 | 1070.41 |  |
| C641 | -0.08 | NS | 0.04 | NS | 4 | 1070.24 |  |
| C642 | -0.01 | NS | 0.08 | NS | 4 | 1119.87 |  |
| C643 | -0.02 | NS | 0 | NS | 4 | 1115.66 |  |
| C644 | 0.05 | NS | 0.10 | NS | 4 | 1146.75 |  |
| **C645** | **0.62** | **0.0024** | 0 | NS | **4** | **700.94** |  |
| C646 | 0.05 | NS | -0.03 | NS | 4 | 702.84 |  |
| C647 | -0.19 | NS | -0.09 | NS | 4 | 723.95 |  |
| C648 | -0.01 | NS | 0.35 | NS | 4 | 803.08 |  |
| C649 | 0.12 | NS | 0.22 | NS | 4 | 790.36 |  |
| C650 | 0 | NS | 0.04 | NS | 4 | 828.18 |  |
| C651 | 0.02 | NS | 0.07 | NS | 4 | 885.61 |  |
| C652 | -0.12 | NS | 0.07 | NS | 4 | 888.14 |  |
| C653 | -0.01 | NS | 0.06 | NS | 4 | 891.2 |  |
| C654 | -0.01 | NS | 0.06 | NS | 4 | 899.3 |  |
| C655 | -1.32 | NS | -0.23 | NS | 4 | 869.17 |  |
| Homoserine | 0.05 | NS | 0.03 | NS | 2 | 537.8 | 335.177 |
| C657 | -0.22 | NS | 0 | NS | 4 | 554.62 |  |
| C658 | -0.23 | NS | -0.14 | NS | 4 | 520.64 |  |
| C659 | 0.53 | NS | -0.22 | NS | 4 | 467.97 |  |
| **C660** | **-0.54** | **0.0224** | 0.04 | NS | **4** | **470.97** |  |
| **C661** | **-0.22** | **0.0007** | 0.02 | NS | **4** | **470.08** |  |
| C662 | -0.10 | NS | 0.02 | NS | 4 | 469.95 |  |
| C663 | -0.13 | NS | -0.08 | NS | 4 | 470.25 |  |
| C664 | 0.19 | NS | -0.11 | NS | 4 | 464.92 |  |
| C665 | 0.16 | NS | 0.25 | NS | 4 | 691.55 |  |
| C666 | -0.71 | NS | 0.05 | NS | 4 | 689.57 |  |
| C667 | -0.47 | NS | 0.13 | NS | 4 | 600.18 |  |
| **C668** | **0.40** | **0.0109** | -0.08 | NS | **4** | **583.23** |  |
| C669 | -0.09 | NS | -0.03 | NS | 4 | 354.64 |  |
| **C670** | **-0.68** | **0.0030** | -0.20 | NS | **4** | **360.46** |  |
| C671 | 0.50 | NS | 0.21 | NS | 4 | 378.03 |  |
| C672 | 0.01 | NS | 0.01 | NS | 4 | 220.87 |  |
| C673 | -0.03 | NS | 0 | NS | 4 | 205.53 |  |
| **C674** | **0.37** | **0.0027** | 0.06 | NS | **4** | **213.85** |  |
| C675 | -0.02 | NS | 0.03 | NS | 4 | 208.43 |  |
| C676 | 0.50 | NS | 0.11 | NS | 4 | 332.47 |  |
| **C677** | **-0.77** | **8.35E-15** | -0.03 | NS | **4** | **319.13** |  |
| **C678** | **-1.37** | **0.0535** | 0.17 | NS | **4** | **322.64** |  |
| C679 | 0.09 | NS | -0.13 | NS | 4 | 322.74 |  |
| C680 | 0.03 | NS | -0.03 | NS | 4 | 325.06 |  |
| C681 | -0.11 | NS | 0.02 | NS | 4 | 932.98 |  |
| C682 | -0.01 | NS | 0.05 | NS | 4 | 915.71 |  |
| C683 | -0.02 | NS | -0.04 | NS | 4 | 968.08 |  |
| C684 | -0.03 | NS | 0.05 | NS | 4 | 978.71 |  |
| C685 | -0.19 | NS | 0.21 | NS | 4 | 1050.62 |  |
| C686 | -0.08 | NS | -0.05 | NS | 4 | 1045.89 |  |
| C687 | 0.04 | NS | 0 | NS | 4 | 1028.57 |  |
| C688 | -0.06 | NS | 0.01 | NS | 4 | 1040.12 |  |
| C689 | -0.24 | NS | -0.11 | NS | 4 | 1038.85 |  |
| C690 | 0.04 | NS | 0.17 | NS | 4 | 1015.51 |  |
| C691 | -0.03 | NS | 0 | NS | 4 | 1018.03 |  |
| C692 | -0.03 | NS | 0.04 | NS | 4 | 997.51 |  |
| C693 | -0.06 | NS | 0.01 | NS | 4 | 1451.26 |  |
| C694 | -0.13 | NS | 0.05 | NS | 4 | 1433.06 |  |
| C695 | 0.14 | NS | -0.08 | NS | 4 | 1446.42 |  |
| C696 | -0.10 | NS | -0.22 | NS | 4 | 1410.18 |  |
| C697 | -0.08 | NS | -0.10 | NS | 4 | 1408.54 |  |
| C698 | -0.02 | NS | 0.05 | NS | 4 | 1431.12 |  |
| C699 | -0.16 | NS | -0.05 | NS | 4 | 1397.41 |  |
| C700 | -0.05 | NS | -0.13 | NS | 4 | 1312.74 |  |
| C701 | -0.12 | NS | 0.11 | NS | 4 | 1306.34 |  |
| C702 | -0.03 | NS | 0.08 | NS | 4 | 1318.7 |  |
| C703 | -0.02 | NS | 0.15 | NS | 4 | 1363.54 |  |
| C704 | -0.10 | NS | -0.14 | NS | 4 | 1368.75 |  |
| C705 | -0.14 | NS | 0.05 | NS | 4 | 1370.68 |  |
| C706 | -0.05 | NS | -0.01 | NS | 4 | 1345.66 |  |
| C707 | 0.13 | NS | 0.04 | NS | 4 | 1345.13 |  |
| C708 | -0.01 | NS | 0.04 | NS | 4 | 1350.57 |  |
| C709 | 0.08 | NS | 0.11 | NS | 4 | 1330.11 |  |
| C710 | -0.07 | NS | -0.13 | NS | 4 | 1505.62 |  |
| C711 | 0.14 | NS | 0.28 | NS | 4 | 1509.34 |  |
| C712 | 0.06 | NS | 0.26 | NS | 4 | 1493.63 |  |
| C713 | 0.01 | NS | 0.01 | NS | 4 | 1462.06 |  |
| C714 | -0.11 | NS | 0.13 | NS | 4 | 1480.7 |  |
| C715 | -0.06 | NS | -0.17 | NS | 4 | 1238.09 |  |
| C716 | -0.20 | NS | 0.01 | NS | 4 | 1212.75 |  |
| C717 | -0.02 | NS | -0.01 | NS | 4 | 1260.84 |  |
| C718 | -0.07 | NS | -0.04 | NS | 4 | 1291.97 |  |
| C719 | -0.03 | NS | 0 | NS | 4 | 1296.06 |  |
| C720 | -0.01 | NS | 0.05 | NS | 4 | 1299.04 |  |
| C721 | -0.14 | NS | -0.01 | NS | 4 | 1284.65 |  |
| C722 | -0.07 | NS | -0.01 | NS | 4 | 1281.45 |  |
| C723 | -0.12 | NS | -0.07 | NS | 4 | 1281.73 |  |
| C724 | -0.02 | NS | 0.11 | NS | 4 | 1272.19 |  |
| C725 | -0.01 | NS | 0.15 | NS | 4 | 1154.84 |  |
| C726 | 0.06 | NS | 0.05 | NS | 4 | 1154.32 |  |
| C727 | -0.06 | NS | 0.14 | NS | 4 | 1152.73 |  |
| C728 | -0.10 | NS | 0.02 | NS | 4 | 1186.4 |  |
| C729 | 0.05 | NS | 0.08 | NS | 4 | 1171.43 |  |
| C730 | 0.13 | NS | 0.02 | NS | 4 | 1168.81 |  |
| C731 | -0.09 | NS | 0.28 | NS | 4 | 1082.82 |  |
| C732 | -0.04 | NS | 0.06 | NS | 4 | 1081.11 |  |
| C733 | 0 | NS | 0.06 | NS | 4 | 1080.6 |  |
| C734 | -0.02 | NS | 0.15 | NS | 4 | 1074.42 |  |
| C735 | -0.04 | NS | -0.05 | NS | 4 | 1059.95 |  |
| C736 | 0 | NS | 0.01 | NS | 4 | 1085.06 |  |
| C737 | -0.11 | NS | 0.10 | NS | 4 | 1103.14 |  |
| C738 | 0.01 | NS | -0.31 | NS | 4 | 1151.23 |  |
| C739 | 0.09 | NS | 0.12 | NS | 4 | 1145.62 |  |
| C740 | -0.06 | NS | 0.09 | NS | 4 | 754.13 |  |
| C741 | -0.03 | NS | 0.08 | NS | 4 | 758.08 |  |
| C742 | -0.18 | NS | -0.39 | NS | 4 | 765.08 |  |
| Putrescine, N-methyl- | 0.05 | NS | 0.07 | NS | 2 | 745.09 | 318.234 |
| C744 | 0.05 | NS | -0.06 | NS | 4 | 715.45 |  |
| C745 | 0.28 | NS | 0.16 | NS | 4 | 736.94 |  |
| C746 | 0.36 | NS | 0.24 | NS | 4 | 721.78 |  |
| C747 | 0.10 | NS | 0.19 | NS | 4 | 731.3 |  |
| C748 | 0.27 | NS | -0.20 | NS | 4 | 793.52 |  |
| C749 | 0.07 | NS | 0.18 | NS | 4 | 828.29 |  |
| C750 | 0.09 | NS | 0.27 | NS | 4 | 835.68 |  |
| C751 | -0.03 | NS | 0.11 | NS | 4 | 881.67 |  |
| C752 | -0.04 | NS | 0.07 | NS | 4 | 851.69 |  |
| C753 | 0.23 | NS | -0.09 | NS | 4 | 853.82 |  |
| C754 | -0.01 | NS | 0.02 | NS | 4 | 861.64 |  |
| C755 | -0.21 | NS | -0.21 | NS | 4 | 573.51 |  |
| C756 | -0.30 | NS | -0.10 | NS | 4 | 532.97 |  |
| **C757** | **0.91** | **0.0002** | 0.16 | NS | **4** | **510.32** |  |
| **C758** | **-0.60** | **0.0415** | 0.01 | NS | **4** | **500** |  |
| C759 | -0.23 | NS | 0.16 | NS | 4 | 447.67 |  |
| **C760** | **-0.53** | **0.0433** | 0.07 | NS | **4** | **485.68** |  |
| C761 | 0.02 | NS | 0.20 | NS | 4 | 684.61 |  |
| C762 | -0.07 | NS | 0.02 | NS | 4 | 681.78 |  |
| C763 | 1.84 | NS | -0.42 | NS | 4 | 665.61 |  |
| C764 | 0.16 | NS | -0.08 | NS | 4 | 596.24 |  |
| C765 | 0.29 | NS | 0.06 | NS | 4 | 601.85 |  |
| C766 | -0.02 | NS | -0.02 | NS | 4 | 589.98 |  |
| **C767** | **-0.62** | **0.0778** | -0.04 | NS | **4** | **651.19** |  |
| C768 | 0.13 | NS | 0.03 | NS | 4 | 659.84 |  |
| C769 | -0.10 | NS | -0.08 | NS | 4 | 616.82 |  |
| C770 | -0.16 | NS | 0 | NS | 4 | 645.01 |  |

ANOVA, analysis of variance; FC, log_2_ fold-change; GC-MS, gas chromatography-mass spectrometry; mass, m/z; PC, phosphatidylcholine; PF, post-farrow; SM, sphingomyelin; WN, weaning.

NS, not significant; ANOVA p-value ≥ 0.2000.

ANOVA p-value ≤ 0.1000, bolded.

^1^Unidentified compound identifier denoted as C###.

^2^Annotation confidence score (scale of 1-4) based on guidelines provided by the Metabolomics Standards Initiative (Sumner et al., 2007).
