# Supplementary material for: Non-targeted Plasma Metabolome of Early and Late Lactation Gilts
Source: Front Mol Biosci. 2016 Nov 24;3:77. doi: 10.3389/fmolb.2016.00077 (PMC5122192; doi:10.3389/fmolb.2016.00077)
Supplement: Supplementary file 2 [file Table2.DOCX]

**Supplementary Table 2. Complete list of 692 compounds or features detected using LC-MS technique with log_2_ fold-change and ANOVA p-value for PF vs WN and Lo loss vs Hi loss effects.**

| **Annotated compound or Feature identifier^1^** | **PF vs WN** | | **Lo vs Hi** | |  |  |  |
| --- | --- | --- | --- | --- | --- | --- | --- |
|  | **FC** | **p-value** | **FC** | **p-value** | **Score^2^** | **RT (sec)** | **m/z** |
| Oleamide | -0.73 | NS | -0.03 | NS | 1 | 356.4194 | 281.272 |
| Ursodeoxycholic acid | -0.86 | NS | 0.41 | NS | 1 | 250.2671 | 392.293 |
| **1-palmitoyl-2-hydroxy-sn-glycero-3-phosphocholine** | **-0.77** | **0.0658** | -0.27 | NS | **1** | **322.553** | **495.332** |
| Chenodeoxycholic acid | -0.62 | NS | 0.29 | NS | 1 | 299.8158 | 392.293 |
| Hyodeoxycholic acid | -0.59 | NS | 0.61 | NS | 1 | 259.3617 | 392.293 |
| C7 | -0.43 | NS | -0.06 | NS | 4 | 632.3208 | 335.1673 |
| 1-oleoyl-2-hydroxy-sn-glycero-3-phosphocholine | -0.67 | 0.1559 | -0.22 | NS | 1 | 337.1274 | 521.348 |
| C9 | 0.07 | NS | 0.02 | NS | 4 | 35.36708 | 695.1001 |
| C10 | 0.04 | NS | 0.04 | NS | 4 | 26.95172 | 214.9182 |
| C11 | -0.02 | NS | -0.12 | NS | 4 | 651.8166 | 478.3296 |
| C12 | 0.22 | NS | 0.11 | NS | 4 | 35.41626 | 294.1011 |
| 1-stearoyl-2-hydroxy-sn-glycero-3-phosphocholine | -0.37 | NS | 0.01 | NS | 1 | 371.4229 | 523.364 |
| C14 | 0.08 | NS | 0.04 | NS | 4 | 29.872 | 288.9225 |
| **lysoPC(18:2)** | **-0.95** | **0.0347** | -0.29 | NS | **2** | **309.5405** | **520.3402** |
| **1-oleoyl-2-palmitoyl-sn-glycero-3-phosphocholine** | **-0.38** | **0.0658** | -0.19 | NS | **1** | **651.6302** | **759.578** |
| lysoPC(20:4) | -0.21 | NS | 0.04 | NS | 2 | 311.5414 | 544.3402 |
| Cholic acid | -0.84 | NS | 0.46 | NS | 2 | 251.3484 | 408.288 |
| PC(34:2) | -0.75 | NS | 0.22 | NS | 2 | 603.7421 | 758.5707 |
| C22 | 0.22 | NS | -0.09 | NS | 4 | 551.7703 | 219.2121 |
| **1,2-dilinolenoyl-sn-glycero-3-phosphocholine** | **-0.56** | **0.0343** | -0.36 | NS | **1** | **632.0548** | **777.531** |
| C24 | 0 | NS | 0.15 | NS | 4 | 35.47269 | 360.0473 |
| M+H=415.2118 | -0.32 | NS | -0.12 | NS | 3 | 227.8172 | 437.1938 |
| C26 | -0.28 | NS | -0.05 | NS | 4 | 660.992 | 335.1674 |
| C27 | 0.28 | NS | 0.10 | NS | 4 | 41.7072 | 161.0641 |
| C28 | 0.16 | NS | 0.10 | NS | 4 | 30.89173 | 363.9308 |
| C29 | 0.03 | NS | -0.19 | NS | 4 | 346.8211 | 518.3223 |
| Hyodeoxycholic acid.1 | -0.59 | NS | 0.47 | NS | 1 | 259.1121 | 392.293 |
| PC(36:2) | -0.18 | NS | -0.10 | NS | 2 | 660.9819 | 786.6035 |
| **PC(40:4)** | **1.01** | **0.0024** | 0.08 | NS | **2** | **682.2406** | **838.6347** |
| C33 | 0.28 | NS | -0.25 | NS | 4 | 675.4196 | 834.6011 |
| Cholestan-3-one, (5α)- | 0.07 | NS | 0.04 | NS | 1 | 552.0569 | 370.3554 |
| L-Tryptophan | -0.17 | NS | -0.03 | NS | 1 | 102.9301 | 204.09 |
| C36 | -0.35 | NS | 0.01 | NS | 4 | 385.9424 | 382.2039 |
| C37 | -0.13 | NS | 0.11 | NS | 4 | 679.1196 | 337.1829 |
| Tryptophan artifact | -0.18 | NS | -0.02 | NS | 1 | 102.8717 | 189.0748 |
| C40 | -0.11 | NS | 0.11 | NS | 4 | 359.5881 | 304.2612 |
| C41 | 1.15 | 0.1943 | -0.19 | NS | 4 | 366.3483 | 265.2535 |
| C42 | -0.56 | NS | 0.47 | NS | 4 | 259.576 | 447.2197 |
| Glycocholic acid | -0.47 | NS | 0.77 | NS | 1 | 222.6393 | 465.309 |
| 16:0 SM (d18:1/16:0) | 0.28 | 0.1943 | 0 | NS | 1 | 612.8183 | 702.568 |
| C45 | -0.08 | NS | -0.02 | NS | 4 | 384.0059 | 563.5516 |
| C46 | -0.28 | NS | -0.17 | NS | 4 | 385.7697 | 226.2176 |
| C47 | 0.36 | NS | 0.03 | NS | 4 | 401.4485 | 526.3772 |
| C48 | -0.02 | NS | -0.13 | NS | 4 | 361.9098 | 281.1547 |
| **1-palmitoyl-2-hydroxy-sn-glycero-3-phosphocholine.1** | **-1.02** | **0.0661** | -0.28 | NS | **1** | **312.4719** | **495.332** |
| Cholic acid.1 | -1.27 | 0.1253 | 0.47 | NS | 1 | 243.749 | 408.288 |
| C51 | -0.14 | NS | -0.26 | NS | 4 | 227.6403 | 453.168 |
| C52 | -0.36 | NS | -0.30 | NS | 4 | 227.8821 | 120.0894 |
| glycochenodeoxycholate-like | -0.66 | NS | 0.79 | NS | 3 | 229.3549 | 414.3005 |
| PE(34:2) | 0.30 | NS | 0.14 | NS | 3 | 613.1278 | 730.5332 |
| C55 | 0.12 | NS | 0.07 | NS | 4 | 26.69455 | 125.9867 |
| C56 | 0.10 | NS | 0.05 | NS | 4 | 26.66464 | 110.0092 |
| C57 | 0.10 | NS | 0.05 | NS | 4 | 28.754 | 486.8685 |
| C58 | 0.39 | NS | 0.25 | NS | 4 | 473.5819 | 339.1985 |
| C59 | -0.17 | NS | 0.03 | NS | 4 | 384.9209 | 283.2831 |
| **1-stearoyl-2-hydroxy-sn-glycero-3-phosphocholine.1** | **1.66** | **0.0988** | 0.16 | NS | **1** | **383.6477** | **523.364** |
| lysoPC(18:0) | 0.12 | NS | -0.28 | NS | 1 | 404.3093 | 524.3717 |
| 1-oleoyl-2-hydroxy-sn-glycero-3-phosphocholine.1 | 1.08 | NS | -0.05 | NS | 1 | 347.4249 | 521.348 |
| C63 | -0.73 | NS | -0.05 | NS | 4 | 357.3558 | 283.2833 |
| 1-oleoyl-2-hydroxy-sn-glycero-3-phosphocholine.2 | 0.02 | NS | -0.04 | NS | 1 | 359.005 | 521.348 |
| **C65** | **-0.85** | **0.0989** | -0.17 | NS | **4** | **322.8579** | **267.6462** |
| **SM(24:1)** | **0.82** | **0.0197** | 0.20 | NS | **3** | **704.4908** | **813.6867** |
| 18:1 SM (d18:1/18:1(9Z)) | 0 | NS | 0.77 | NS | 1 | 594.0495 | 728.583 |
| 1,2-dilinoleoyl-sn-glycero-3-phosphocholine | -1.19 | 0.1943 | 0.05 | NS | 1 | 591.6043 | 781.562 |
| C69 | 0.25 | NS | -0.15 | NS | 4 | 623.2106 | 808.5838 |
| C70 | 0.35 | NS | -0.05 | NS | 4 | 624.0589 | 729.5923 |
| PC(38:4) | 0.83 | NS | -0.06 | NS | 2 | 655.8827 | 810.6027 |
| C72 | -0.28 | NS | -0.09 | NS | 4 | 634.3665 | 797.5305 |
| C73 | -0.09 | NS | -0.05 | NS | 4 | 633.8405 | 184.074 |
| C74 | -0.21 | NS | -0.25 | NS | 4 | 632.5319 | 780.5538 |
| C75 | 0.15 | NS | 0.09 | NS | 4 | 31.2928 | 226.9521 |
| C76 | 0.13 | NS | 0.12 | NS | 4 | 29.8547 | 272.945 |
| Palmitic acid | 0.49 | NS | -0.10 | NS | 1 | 414.6776 | 256.24 |
| C78 | -0.35 | NS | -0.21 | NS | 4 | 384.4878 | 284.2862 |
| C79 | 0.31 | NS | -0.03 | NS | 4 | 378.2482 | 305.2644 |
| 1-stearoyl-2-hydroxy-sn-glycero-3-phosphocholine.2 | 0.39 | NS | 0.09 | NS | 1 | 397.2794 | 523.364 |
| C81 | 1.06 | NS | 0.24 | NS | 4 | 396.9311 | 335.1672 |
| C82 | 0.27 | NS | 0.04 | NS | 4 | 406.2276 | 638.3099 |
| C83 | 0.20 | NS | -0.23 | NS | 4 | 347.5861 | 104.1076 |
| C84 | 0 | NS | -0.36 | NS | 4 | 348.422 | 497.3438 |
| Linoleic acid | -0.16 | NS | 0.24 | NS | 2 | 370.224 | 280.24 |
| C86 | -0.26 | NS | -0.32 | NS | 4 | 365.165 | 523.3596 |
| C87 | 0.37 | NS | 0 | NS | 4 | 365.7724 | 319.1949 |
| C88 | -0.39 | NS | -0.22 | NS | 4 | 328.9867 | 254.2486 |
| C89 | -0.83 | 0.1943 | 0.61 | NS | 4 | 223.3104 | 414.3004 |
| C90 | -0.31 | NS | 0.13 | NS | 4 | 582.1113 | 372.2617 |
| 16:0 SM (d18:1/16:0).1 | -0.12 | NS | 0.42 | NS | 1 | 582.2109 | 702.568 |
| C92 | 0.20 | NS | -0.02 | NS | 4 | 662.5577 | 184.0741 |
| C93 | 0.42 | NS | 0.16 | NS | 4 | 667.3889 | 385.1841 |
| C94 | 0.51 | 0.1425 | -0.08 | NS | 4 | 667.4746 | 858.6008 |
| C95 | 0.20 | NS | 0.22 | NS | 4 | 613.1453 | 370.2436 |
| **C96** | **0.51** | **0.0284** | 0 | NS | **4** | **654.8762** | **768.5915** |
| **18:0 SM (d18:1/18:0)** | **0.62** | **0.0603** | 0.25 | NS | **1** | **644.8523** | **730.599** |
| PC(36:2).1 | -0.12 | NS | 0.41 | NS | 2 | 640.4361 | 786.5941 |
| Cholestan-3-one, (5α)-.1 | 0.20 | NS | -0.02 | NS | 2 | 551.2082 | 369.352 |
| C100 | 0.07 | NS | 0.02 | NS |  | 40.44978 | 293.0984 |
| Monosaccharide | 0.05 | NS | 0.08 | NS | 3 | 34.50367 | 203.0534 |
| C102 | -0.21 | NS | 0.30 | NS | 4 | 32.139 | 470.9957 |
| C103 | 0.09 | NS | -0.10 | NS | 4 | 30.58444 | 304.8966 |
| C104 | 0.21 | NS | -0.02 | NS | 4 | 400.826 | 543.3414 |
| C105 | 0.13 | NS | -0.52 | NS | 4 | 346.0325 | 515.3104 |
| 1-stearoyl-2-hydroxy-sn-glycero-3-phosphocholine.3 | -0.34 | NS | -0.02 | NS | 1 | 359.9546 | 523.364 |
| 1-palmitoyl-2-hydroxy-sn-glycero-3-phosphocholine.2 | 1.14 | 0.1827 | -0.36 | NS | 1 | 334.108 | 495.332 |
| lysoPC(18:2).1 | -0.28 | NS | -0.33 | NS | 2 | 332.2793 | 520.34 |
| C109 | -0.15 | NS | -0.20 | NS | 4 | 328.5125 | 156.1393 |
| C110 | -1.37 | NS | 0.10 | NS | 4 | 282.8076 | 358.2823 |
| C111 | -0.18 | NS | 0.12 | NS | 4 | 250.5269 | 281.1734 |
| **C112** | **0.48** | **0.0074** | 0.28 | 0.1853 | **4** | **704.1198** | **434.3035** |
| C113 | 0 | NS | 0.37 | NS | 4 | 606.8534 | 410.7597 |
| C114 | 0.32 | 0.1331 | 0.13 | NS | 4 | 664.4194 | 848.5612 |
| PC(38:4).1 | 0.42 | 0.1559 | 0.16 | NS | 2 | 664.3018 | 810.6034 |
| C16-20:4 PC | -0.25 | NS | 0 | NS | 2 | 669.6776 | 767.583 |
| 1,2-dioleoyl-sn-glycero-3-phosphocholine | -0.23 | NS | 0.07 | NS | 1 | 679.2961 | 785.593 |
| C118 | -0.17 | NS | 0.20 | NS | 4 | 678.9878 | 506.3609 |
| PC(38:3) | -0.06 | NS | 0 | NS | 2 | 675.6841 | 812.6176 |
| C120 | 0.16 | NS | 0.23 | NS | 4 | 613.3215 | 379.7514 |
| **C121** | **0.37** | **0.0995** | 0 | NS | **4** | **612.7074** | **705.5839** |
| **PC(O-34:3)** | **-0.53** | **0.0356** | 0.02 | NS | **2** | **650.9358** | **742.5767** |
| C123 | 0.31 | NS | 0.02 | NS | 4 | 644.9451 | 808.5865 |
| **C124** | **-0.64** | **0.0811** | -0.10 | NS | **4** | **641.4156** | **335.1676** |
| C125 | 0.08 | NS | -0.01 | NS | 4 | 34.78988 | 116.0711 |
| C126 | 0.24 | NS | 0.14 | NS | 4 | 35.59613 | 114.0624 |
| C127 | 0.16 | NS | 0.07 | NS | 4 | 31.865 | 104.9928 |
| C128 | 0.60 | 0.1559 | 0.22 | NS | 4 | 416.4834 | 342.1862 |
| C129 | 0.38 | NS | 0.24 | NS | 4 | 415.966 | 311.1671 |
| C130 | 1.78 | 0.1601 | 0.16 | NS | 4 | 430.9971 | 619.4381 |
| C131 | -0.22 | NS | -0.21 | NS | 4 | 385.5389 | 282.2799 |
| C132 | 0.87 | NS | -0.07 | NS | 4 | 378.7519 | 335.1886 |
| C133 | 1.17 | NS | 0.16 | NS | 4 | 398.3889 | 603.4667 |
| C134 | 0.18 | NS | -0.63 | NS | 4 | 345.5789 | 1014.663 |
| **C135** | **-0.62** | **0.0543** | -0.24 | NS | **4** | **327.0681** | **522.347** |
| C136 | -0.57 | NS | 0.06 | NS | 4 | 302.509 | 605.4248 |
| **lysoPC(18:2).2** | **-1.13** | **0.0311** | -0.34 | NS | **2** | **300.192** | **520.3399** |
| C138 | -0.86 | NS | 0.39 | NS | 4 | 251.3239 | 463.2154 |
| Alpha-Tocopherol | -0.14 | NS | 0.03 | NS | 1 | 590.5211 | 430.381 |
| C140 | 1.66 | NS | 0.30 | NS | 4 | 592.5549 | 704.579 |
| C141 | -0.49 | 0.1446 | -0.05 | NS | 4 | 658.8351 | 504.3453 |
| C142 | -0.27 | NS | 0.21 | NS | 4 | 669.2 | 746.6075 |
| PC(38:4).2 | -0.01 | NS | -0.01 | NS | 2 | 679.1946 | 810.6008 |
| C144 | 0.12 | NS | 0.53 | NS | 4 | 614.2701 | 808.5855 |
| C145 | 0.47 | NS | -0.40 | NS | 4 | 621.097 | 410.76 |
| C146 | -0.29 | NS | 0.36 | NS | 4 | 626.0286 | 321.2106 |
| **PC(36:2).2** | **-1.29** | **0.0007** | -0.59 | 0.1658 | **2** | **647.313** | **772.5867** |
| PC(38:5) | 0.17 | NS | -0.04 | NS | 2 | 638.7796 | 808.5865 |
| 1,2-dioleoyl-sn-glycero-3-phosphocholine.1 | -0.19 | NS | 0.48 | NS | 1 | 638.5653 | 785.593 |
| PC(38:4).3 | 0.52 | NS | 0.75 | NS | 2 | 640.5131 | 810.5939 |
| L-Tyrosine | -0.42 | NS | 0.05 | NS | 1 | 45.04814 | 181.074 |
| C152 | 0.14 | NS | 0.05 | NS | 4 | 31.40343 | 242.926 |
| C153 | 0.07 | NS | 0.07 | NS | 4 | 28.78743 | 402.9032 |
| **Hippuric acid** | **-1.28** | **0.0007** | -0.08 | NS | **1** | **116.7797** | **179.058** |
| **C155** | **0.88** | **0.0658** | **0.91** | **0.0565** | **4** | **432.5588** | **337.1823** |
| C156 | -0.24 | NS | -0.24 | NS | 4 | 383.4857 | 583.0257 |
| C157 | -0.13 | NS | 0.07 | NS | 4 | 383.6628 | 551.4344 |
| C158 | 0.45 | NS | -0.04 | NS | 4 | 350.1963 | 280.2641 |
| C159 | -0.52 | NS | -0.09 | NS | 4 | 355.503 | 135.1176 |
| C160 | 0.11 | NS | 0.05 | NS | 4 | 356.4367 | 141.9715 |
| C161 | -0.23 | NS | -0.08 | NS | 4 | 310.8382 | 228.233 |
| C162 | 0 | NS | 0.14 | NS | 4 | 322.5135 | 605.425 |
| lysoPC(20:4).1 | 1.53 | 0.1060 | **0.06** | **NS** | 2 | 320.3828 | 544.3397 |
| **C164** | **0.80** | **0.0296** | **-0.01** | **NS** | **4** | **333.2727** | **291.646** |
| C165 | -0.16 | NS | **-0.20** | **NS** | 4 | 330.3265 | 279.6459 |
| C166 | -0.59 | NS | **-0.36** | **NS** | 4 | 327.9282 | 1061.663 |
| C167 | -0.89 | NS | **0.13** | **NS** | 4 | 283.1152 | 357.2789 |
| C168 | 0.03 | NS | **0.10** | **NS** | 4 | 262.5673 | 301.1416 |
| 3a,6b,7b-Trihydroxy-5b-cholanoic acid | -0.79 | NS | **0.53** | **NS** | 1 | 251.3208 | 408.288 |
| C170 | -0.03 | NS | **0.15** | **NS** | 4 | 220.4562 | 263.057 |
| **C171** | **0.63** | **0.0014** | **-0.13** | **NS** | **4** | **596.4142** | **689.5615** |
| PC(36:2).3 | -0.07 | NS | **0.16** | **NS** | 2 | 661.3883 | 788.6097 |
| C173 | -0.20 | NS | **-0.03** | **NS** | 4 | 662.6483 | 507.3648 |
| C174 | 0.42 | 0.1787 | **0.08** | **NS** | 4 | 664.3882 | 424.7753 |
| C175 | -0.12 | NS | **-0.20** | **NS** | 4 | 660.8948 | 808.5851 |
| **C176** | **0.43** | **0.0005** | **0.10** | **NS** | **4** | **687.1417** | **143.9597** |
| C177 | 0.44 | NS | 0.10 | NS | 4 | 685.0803 | 814.6355 |
| C178 | 0.38 | NS | 0.09 | NS | 4 | 679.4763 | 794.6085 |
| **C179** | **1.04** | **0.0004** | 0.07 | NS | **4** | **682.4455** | **438.791** |
| C180 | -0.86 | NS | -1.66 | NS | 4 | 614.8883 | 503.1087 |
| C181 | 0.33 | 0.1943 | 0.06 | NS | 4 | 613.0523 | 461.2935 |
| C182 | 0.41 | NS | 0.05 | NS | 4 | 612.432 | 722.5458 |
| C183 | -0.12 | NS | -0.60 | 0.1509 | 4 | 621.3818 | 804.5536 |
| C184 | 0.37 | NS | 0.09 | NS | 4 | 624.8207 | 392.2572 |
| **PC(32:2)** | **-0.60** | **0.0345** | -0.39 | NS | **2** | **622.7085** | **732.5558** |
| C186 | 0.55 | 0.1426 | 0.06 | NS | 4 | 624.6108 | 705.5922 |
| C187 | 1.40 | NS | -0.14 | NS | 4 | 654.2967 | 836.6182 |
| **C188** | **-0.57** | **0.0522** | -0.24 | NS | **4** | **651.5105** | **1159.849** |
| C189 | 0.11 | NS | -0.06 | NS | 4 | 634.9628 | 798.5334 |
| C190 | -0.05 | NS | -0.23 | NS | 4 | 633.2765 | 802.0422 |
| C191 | -0.05 | NS | -0.04 | NS | 4 | 631.9523 | 406.747 |
| **C192** | **-0.51** | **0.0569** | **-0.49** | **0.0737** | **4** | **641.1008** | **803.5552** |
| **PC(36:3)** | **-0.64** | **0.0007** | -0.31 | 0.1559 | **2** | **641.6088** | **784.5871** |
| C194 | -0.10 | NS | -0.04 | NS | 4 | 642.1598 | 504.3452 |
| C195 | 0.14 | NS | -0.02 | NS | 4 | 551.4738 | 371.3589 |
| C196 | 0.21 | NS | -0.01 | NS | 4 | 550.78 | 287.2746 |
| C197 | 0.03 | NS | 0.20 | NS | 4 | 557.1177 | 701.5605 |
| C198 | -0.04 | NS | -0.01 | NS | 4 | 1079.176 | 214.9182 |
| C199 | 0.18 | NS | 0.17 | NS | 4 | 41.45583 | 294.1013 |
| C200 | 0.01 | NS | 0.04 | NS | 4 | 41.40967 | 317.0839 |
| C201 | 0.19 | NS | 0.21 | NS | 4 | 36.471 | 257.0748 |
| C202 | -0.06 | NS | 0.02 | NS | 4 | 33.4115 | 80.9485 |
| C203 | 0.11 | NS | 0.08 | NS | 4 | 26.64633 | 199.9424 |
| C204 | 0.07 | NS | 0.07 | NS | 4 | 32.13133 | 459.9413 |
| C205 | 0.17 | NS | 0.10 | NS | 4 | 30.797 | 1039.792 |
| C206 | 0.11 | NS | 0.07 | NS | 4 | 29.20117 | 606.8659 |
| C207 | 0.14 | NS | 0.06 | NS | 4 | 30.89733 | 884.8367 |
| C208 | 0.04 | NS | 0.17 | NS | 4 | 109.5883 | 235.0938 |
| C209 | -0.09 | NS | 0.01 | NS | 4 | 98.819 | 302.1968 |
| **C210** | **-1.70** | **0.0075** | 0.09 | NS | **4** | **121.8413** | **340.104** |
| **C211** | **-1.68** | **0.0004** | -0.30 | NS | **4** | **127.3597** | **431.0976** |
| C212 | 0.40 | NS | -0.14 | NS | 4 | 472.6406 | 323.2264 |
| C213 | 0.89 | NS | -0.18 | NS | 4 | 413.8642 | 551.4349 |
| C214 | -0.36 | NS | 0.29 | NS | 4 | 437.7594 | 323.226 |
| C215 | 2.38 | NS | -0.38 | NS | 4 | 430.0194 | 603.4671 |
| C216 | -0.44 | NS | -0.33 | NS | 4 | 384.642 | 663.4782 |
| C217 | -0.47 | NS | 0.26 | NS | 4 | 384.2684 | 295.1949 |
| C218 | 0.24 | NS | 0.56 | NS | 4 | 399.025 | 321.2106 |
| C219 | 0.22 | NS | 0.01 | NS | 4 | 404.2822 | 289.6483 |
| C220 | 0 | NS | -0.05 | NS | 4 | 347.6894 | 312.0294 |
| C221 | -0.23 | NS | -0.17 | NS | 4 | 346.6308 | 266.6274 |
| C222 | -0.46 | NS | -0.04 | NS | 4 | 367.0216 | 256.2643 |
| C223 | -0.28 | NS | -0.60 | NS | 4 | 364.6252 | 545.3419 |
| Biliverdin hydrochloride | 1.76 | NS | -0.77 | NS | 1 | 309.902 | 582.248 |
| C225 | 0.03 | NS | -0.17 | NS | 4 | 323.9418 | 649.4517 |
| C226 | -1.20 | NS | -0.29 | NS | 4 | 340.1806 | 256.2641 |
| **C227** | **0.77** | **0.0356** | -0.16 | NS | **4** | **332.4534** | **268.1523** |
| C228 | 0.35 | NS | 0.01 | NS | 4 | 327.0556 | 544.3397 |
| C229 | 0.13 | NS | 0.10 | NS | 4 | 286.3106 | 306.0922 |
| Glycocholic acid.1 | -0.93 | NS | 0.99 | NS | 1 | 217.1312 | 465.309 |
| Cortexolone | 0.39 | NS | 0.12 | NS | 4 | 204.4022 | 346.214 |
| C232 | 0.13 | NS | 0.08 | NS | 4 | 203.052 | 274.2747 |
| C233 | -0.85 | 0.1317 | -0.26 | NS | 4 | 606.086 | 478.3294 |
| C234 | 0.02 | NS | 0.45 | NS | 4 | 606.3774 | 783.5748 |
| C235 | 0.30 | 0.1639 | 0.08 | NS | 4 | 590.423 | 378.2411 |
| C236 | 0.22 | NS | -0.26 | NS | 4 | 578.825 | 675.545 |
| C237 | 0.66 | 0.1819 | 0.30 | NS | 4 | 586.0314 | 725.5582 |
| C238 | -0.40 | NS | 0.16 | NS | 4 | 658.1178 | 321.211 |
| **C239** | **0.68** | **0.0653** | 0.39 | NS | **4** | **664.061** | **838.062** |
| **C16-20:3 PC** | **-0.73** | **0.0024** | 0.02 | NS | **1** | **659.0428** | **769.599** |
| **C241** | **-1.58** | **0.0015** | -0.48 | NS | **4** | **665.8848** | **774.6028** |
| C242 | 0.51 | 0.1658 | 0.07 | NS | 4 | 666.9742 | 836.6185 |
| C243 | 1.32 | NS | 0.72 | NS | 4 | 687.4076 | 813.684 |
| **C244** | **0.89** | **0.0027** | 0.03 | NS | **4** | **687.08** | **811.6657** |
| 1-(1Z-octadecenyl)-2-oleoyl-sn-glycero-3-phosphocholine | -0.40 | 0.1890 | 0.07 | NS | 1 | 678.334 | 771.614 |
| C246 | 0.72 | 0.1943 | -0.05 | NS | 4 | 672.8366 | 796.6231 |
| C247 | 0.07 | NS | -0.37 | NS | 4 | 676.2028 | 685.4396 |
| PC(36:5) | 0.22 | NS | -0.25 | NS | 2 | 616.9508 | 780.5549 |
| **C249** | **-1.09** | **0.0039** | -0.65 | 0.1272 | **4** | **617.21** | **464.3148** |
| **C250** | **-0.66** | **0.0296** | -0.26 | NS | **4** | **616.1162** | **802.537** |
| **1,2-dioleoyl-sn-glycero-3-phosphoethanolamine** | **-1.54** | **0.0011** | -0.66 | NS | **1** | **616.9482** | **743.547** |
| PC(36:3).1 | -0.51 | NS | -0.04 | NS | 2 | 613.9052 | 784.5861 |
| C253 | -0.44 | 0.1658 | -0.33 | NS | 4 | 612.8222 | 778.5394 |
| C254 | 0.22 | NS | -0.03 | NS | 4 | 612.9762 | 788.5262 |
| C255 | 0.36 | 0.1943 | 0.04 | NS | 4 | 612.1918 | 706.5795 |
| **C256** | **-0.71** | **0.0075** | -0.45 | 0.1437 | **4** | **621.5678** | **502.3296** |
| C257 | -0.39 | NS | -0.51 | 0.1051 | 4 | 621.561 | 820.5318 |
| PC(36:3).2 | 0.01 | NS | 0.54 | NS | 2 | 621.8292 | 784.5791 |
| **C259** | **-1.40** | **0.0005** | -0.54 | NS | **4** | **626.724** | **770.5705** |
| C260 | -0.28 | NS | 0.50 | NS | 4 | 627.7898 | 400.2689 |
| C261 | -0.33 | NS | 0.15 | NS | 4 | 652.0304 | 744.592 |
| C262 | -0.06 | NS | -0.18 | NS | 4 | 651.4598 | 321.2107 |
| C263 | 0 | NS | -0.13 | NS | 4 | 652.0444 | 782.5694 |
| C264 | 0.81 | NS | 0.92 | NS | 4 | 644.9328 | 836.6181 |
| PC(36:2).4 | 0.95 | NS | -0.11 | NS | 2 | 647.6262 | 786.5956 |
| **C266** | **-0.53** | **0.0536** | -0.27 | NS | **4** | **647.169** | **361.1822** |
| **C267** | **-0.70** | **0.0813** | -0.32 | NS | **4** | **648.9142** | **796.5864** |
| **PC(36:3).3** | **-0.73** | **0.0255** | -0.38 | NS | **2** | **648.0042** | **784.5853** |
| 1-oleoyl-2-palmitoyl-sn-glycero-3-phosphocholine.1 | -0.07 | NS | 0.01 | NS | 1 | 632.305 | 759.578 |
| C270 | 0.23 | NS | 0.12 | NS | 4 | 635.6778 | 359.1672 |
| C271 | 0.24 | NS | -0.03 | NS | 4 | 635.2914 | 805.5578 |
| C272 | 0.11 | NS | -0.15 | NS | 4 | 635.1384 | 782.5713 |
| C273 | 0.04 | NS | -0.19 | NS | 4 | 633.8206 | 1171.836 |
| **C274** | **-0.53** | **0.0087** | -0.28 | NS | **4** | **632.759** | **758.5724** |
| C275 | -0.15 | NS | -0.13 | NS | 4 | 640.2918 | 412.2699 |
| C276 | 0.34 | NS | 0.14 | NS | 4 | 642.5002 | 734.5725 |
| C277 | 0.31 | NS | 0.31 | NS | 4 | 638.8238 | 385.1831 |
| C278 | 0.28 | NS | 0.17 | NS | 4 | 37.6844 | 315.0804 |
| C279 | -0.30 | 0.1080 | 0.09 | NS | 4 | 35.669 | 138.054 |
| C280 | 0.15 | NS | 0.05 | NS | 4 | 35.5884 | 164.9301 |
| C281 | 0.01 | NS | 0.03 | NS | 4 | 35.9784 | 396.997 |
| C282 | 0.10 | NS | 0.08 | NS | 4 | 32.489 | 255.9786 |
| C283 | 0.23 | NS | 0.12 | NS | 4 | 31.8392 | 327.0195 |
| C284 | -0.34 | NS | -0.01 | NS | 4 | 29.3556 | 177.1238 |
| C285 | 0.19 | NS | -0.02 | NS | 4 | 31.567 | 122.9253 |
| C286 | -0.07 | NS | 0.19 | NS | 4 | 55.2984 | 293.0984 |
| C287 | 0.21 | NS | 0.24 | NS | 4 | 474.6075 | 340.2022 |
| C288 | 0.13 | NS | 0.13 | NS | 4 | 416.0735 | 295.195 |
| C289 | 0.04 | NS | 0.08 | NS | 4 | 442.4288 | 341.2452 |
| C290 | -0.12 | NS | -0.20 | NS | 4 | 387.5805 | 305.2642 |
| C291 | 1.71 | 0.1342 | 0.10 | NS | 4 | 382.3463 | 547.3571 |
| C292 | 0.18 | NS | 0.06 | NS | 4 | 375.9418 | 482.3591 |
| C293 | 0.53 | NS | 0.17 | NS | 4 | 396.9523 | 659.2883 |
| C294 | 1.56 | 0.1943 | -0.11 | NS | 4 | 395.1568 | 303.2266 |
| C295 | 0.17 | NS | 0.28 | NS | 4 | 404.2465 | 637.3061 |
| C296 | 0.10 | NS | -0.08 | NS | 4 | 403.057 | 281.6615 |
| C297 | 0.30 | NS | 0.01 | NS | 4 | 347.5015 | 302.2459 |
| C298 | 0.29 | NS | -0.25 | NS | 4 | 346.4365 | 157.5367 |
| C299 | 0.09 | NS | -0.19 | NS | 4 | 346.6683 | 523.296 |
| C300 | 0.17 | NS | -0.98 | NS | 4 | 344.0898 | 762.9796 |
| C301 | 0.07 | NS | -0.05 | NS | 4 | 370.8158 | 146.9825 |
| 1-oleoyl-2-hydroxy-sn-glycero-3-phosphocholine.3 | 0.61 | NS | -0.11 | NS | 1 | 352.924 | 521.348 |
| C303 | -0.60 | NS | -0.30 | NS | 4 | 355.8285 | 269.2679 |
| C304 | -0.09 | NS | 0.12 | NS | 4 | 361.5783 | 487.3613 |
| C305 | 0 | NS | 0 | NS | 4 | 365.0078 | 279.2479 |
| C306 | -0.12 | NS | -0.25 | NS | 4 | 309.4125 | 254.2486 |
| C307 | 0.84 | NS | -0.07 | NS | 4 | 318.4368 | 521.3437 |
| C308 | -0.15 | NS | 0.13 | NS | 4 | 336.7023 | 561.4 |
| C309 | 0.06 | NS | -0.21 | NS | 4 | 331.3843 | 104.1077 |
| C310 | 1.30 | 0.1996 | -0.16 | NS | 4 | 333.9968 | 519.3263 |
| C311 | -0.25 | NS | -0.43 | NS | 4 | 332.9108 | 478.298 |
| C312 | 0.36 | NS | -0.21 | NS | 4 | 332.213 | 568.3373 |
| C313 | -0.44 | NS | -0.13 | NS | 4 | 331.289 | 278.6269 |
| C314 | -0.18 | NS | -0.22 | NS | 4 | 328.672 | 149.1333 |
| C315 | 0 | NS | -0.15 | NS | 4 | 328.4908 | 198.1862 |
| C316 | -0.01 | NS | -0.27 | NS | 4 | 295.8623 | 468.31 |
| C317 | -1.09 | 0.1034 | -0.98 | 0.1559 | 4 | 293.299 | 494.3248 |
| C318 | -0.48 | NS | 0.36 | NS | 4 | 259.2205 | 451.2195 |
| **C319** | **-1.22** | **0.0356** | 0.87 | 0.1688 | **4** | **249.579** | **391.2849** |
| C320 | -0.64 | NS | 0.30 | NS | 4 | 252.7715 | 414.3008 |
| C321 | -0.49 | NS | -0.47 | NS | 4 | 226.9783 | 169.0502 |
| C322 | -0.15 | NS | -0.18 | NS | 4 | 227.9125 | 460.2702 |
| Deoxycorticosterone | 0.26 | NS | 0.07 | NS | 1 | 204.2685 | 330.219 |
| **C324** | **0.99** | **0.0214** | 0.28 | NS | **4** | **703.779** | **815.6955** |
| C325 | 0.71 | NS | 0.32 | NS | 4 | 701.1468 | 787.6697 |
| 24:0 SM | 0.91 | 0.1658 | 0.34 | NS | 1 | 726.6513 | 814.693 |
| **C327** | **0.25** | **0.0989** | 0.13 | NS | **4** | **712.9393** | **365.1061** |
| C328 | -0.62 | NS | -0.04 | NS | 4 | 604.1295 | 479.3329 |
| C329 | 0.87 | NS | -0.01 | NS | 4 | 605.0125 | 806.5686 |
| **C330** | **0.79** | **0.0004** | -0.20 | NS | **4** | **589.3878** | **370.2522** |
| C331 | 1.17 | NS | 0.10 | NS | 4 | 595.5683 | 705.5893 |
| C332 | -0.09 | NS | 0.51 | NS | 4 | 659.7185 | 790.6238 |
| **C333** | **1.13** | **0.0087** | 0.32 | NS | **4** | **659.7073** | **834.6** |
| C334 | -0.31 | 0.1113 | -0.19 | NS | 4 | 660.9478 | 815.0642 |
| C335 | 0.40 | NS | 0.11 | NS | 4 | 660.773 | 794.6078 |
| **C336** | **0.96** | **0.0465** | 0.16 | NS | **4** | **666.8398** | **838.6256** |
| C337 | 0.03 | NS | -0.06 | NS | 4 | 679.0338 | 321.2108 |
| C338 | 0.26 | NS | 0.20 | NS | 4 | 673.4548 | 365.106 |
| **C339** | **0.82** | **0.0087** | 0.21 | NS | **4** | **673.581** | **759.6401** |
| C340 | 0.88 | NS | 0.05 | NS | 4 | 615.091 | 758.5651 |
| PC(32:2).1 | 0.34 | NS | -0.34 | NS | 2 | 613.1565 | 732.542 |
| C342 | -0.30 | NS | -0.27 | NS | 4 | 613.7963 | 756.5549 |
| C343 | 0.23 | NS | 0.04 | NS | 4 | 613.169 | 184.074 |
| C344 | -0.66 | 0.1658 | -0.47 | NS | 4 | 621.78 | 335.1673 |
| **C345** | -0.57 | 0.1253 | **-0.77** | **0.0267** | **4** | **621.778** | **599.5044** |
| C346 | -1.57 | 0.1943 | 0.06 | NS | 4 | 621.0865 | 772.5871 |
| **C347** | **0.77** | **0.0196** | 0.20 | NS | **4** | **655.597** | **406.2729** |
| **C348** | **0.62** | **0.0470** | 0.06 | NS | **4** | **647.1078** | **834.6004** |
| **C349** | **1.56** | **0.0527** | -0.06 | NS | **4** | **649.5648** | **832.5844** |
| C350 | 0.63 | NS | 0.07 | NS | 4 | 631.4595 | 806.5692 |
| C351 | -0.22 | NS | -0.13 | NS | 4 | 630.419 | 1159.842 |
| C352 | 0.75 | NS | 0.27 | NS | 4 | 630.199 | 810.5966 |
| C353 | 0.34 | 0.1658 | 0.09 | NS | 4 | 635.6923 | 820.5318 |
| **C354** | **0.36** | **0.0656** | -0.07 | NS | **4** | **635.3513** | **804.5539** |
| **C355** | **1.31** | **0.0658** | 0.62 | NS | **4** | **631.0653** | **537.3962** |
| Phosphatidylethanolamine-ceramide(d16:1/22:0) | 0.20 | NS | -0.11 | NS | 2 | 628.8993 | 717.5941 |
| C357 | 0.34 | NS | 0.26 | NS | 4 | 643.6083 | 359.1678 |
| C358 | -0.23 | NS | 0.04 | NS | 4 | 641.6768 | 505.3492 |
| **C359** | **0.36** | **0.0434** | -0.02 | NS | **4** | **638.9603** | **846.5466** |
| **C360** | **-1.45** | **0.0023** | -0.68 | NS | **4** | **637.3503** | **746.5728** |
| C361 | -0.79 | NS | -0.07 | NS | 4 | 638.608 | 412.7746 |
| Cholesterol-like | 0.12 | NS | 0.78 | NS | 3 | 517.642 | 386.355 |
| C363 | 0.08 | NS | 0.15 | NS | 4 | 746.998 | 97.9694 |
| C364 | 0.17 | NS | 0.01 | NS | 4 | 43.51475 | 331.05 4 |
| C365 | 0.07 | NS | 0.15 | NS | 4 | 41.0645 | 651.1355 |
| C366 | -0.03 | NS | 0.08 | NS | 4 | 33.9445 | 365.1056 |
| L-Acetylcarnitine | 0.96 | NS | -0.97 | NS | 1 | 35.371 | 203.116 |
| C368 | 0.33 | NS | 0.08 | NS | 4 | 37.278 | 324.0028 |
| **C369** | **0.43** | **0.0658** | 0.11 | NS | **4** | **35.7815** | **189.0873** |
| C370 | 0.24 | NS | 0.13 | NS | 4 | 33.82475 | 130.0508 |
| C371 | -0.27 | NS | 0.08 | NS | 4 | 34.40025 | 460.9672 |
| C372 | 0.19 | NS | 0.01 | NS | 4 | 35.19475 | 202.1808 |
| C373 | -0.02 | NS | 0.03 | NS | 4 | 26.3045 | 101.0089 |
| C374 | 0.08 | NS | 0.24 | NS | 4 | 32.65475 | 134.0196 |
| C375 | 0.20 | NS | 0.15 | NS | 4 | 32.82925 | 191.0413 |
| C376 | 0.03 | NS | 0.08 | NS | 4 | 31.328 | 355.0584 |
| L-Leucine | 0 | NS | 0.14 | NS | 1 | 58.1175 | 131.095 |
| C378 | 0.10 | NS | 0.02 | NS | 4 | 124.1173 | 182.1912 |
| **Caprolactam** | **-3.65** | **0.0257** | 0.58 | NS | **1** | **115.0588** | **113.084** |
| Hesperidin | 0.10 | NS | 0.36 | NS | 1 | 133.8918 | 610.19 |
| C381 | -0.50 | NS | -0.38 | NS | 4 | 416.962 | 284.2955 |
| Stearamide | -0.55 | NS | -0.19 | NS | 2 | 425.57 | 283.288 |
| C383 | -0.44 | NS | 0.12 | NS | 4 | 421.916 | 413.2663 |
| C384 | 0.09 | NS | -0.16 | NS | 4 | 450.0527 | 413.2666 |
| 7-ketocholesterol | -0.41 | NS | -0.13 | NS | 1 | 446.6583 | 401.3422 |
| C386 | 1.12 | NS | 0.45 | NS | 4 | 438.079 | 332.2927 |
| C387 | 1.32 | 0.1076 | 0.48 | NS | 4 | 434.955 | 327.2273 |
| C388 | 2.54 | 0.1559 | -0.05 | NS | 4 | 432.9217 | 618.4319 |
| 1-stearoyl-2-hydroxy-sn-glycero-3-phosphocholine.4 | 0.86 | NS | 0.27 | NS | 1 | 390.7033 | 523.364 |
| C390 | -0.25 | NS | -0.45 | NS | 4 | 384.2497 | 585.5349 |
| C391 | -0.15 | NS | -0.29 | NS | 4 | 384.971 | 198.1862 |
| C392 | -0.40 | NS | -0.11 | NS | 4 | 387.437 | 270.2799 |
| C393 | 0.79 | NS | -0.25 | NS | 4 | 385.2277 | 334.1866 |
| C394 | -0.43 | NS | -0.05 | NS | 4 | 381.7607 | 339.2246 |
| C395 | -0.62 | NS | -0.18 | NS | 4 | 391.9673 | 284.2955 |
| lysoPC(20:3) | 0.59 | NS | 0.39 | NS | 2 | 394.8293 | 546.354 |
| C397 | 2.55 | 0.1996 | -0.01 | NS | 4 | 401.034 | 604.4711 |
| C398 | 0.12 | NS | 0.14 | NS | 4 | 405.2693 | 660.2921 |
| C399 | 0.56 | NS | 0.37 | NS | 4 | 402.152 | 607.7958 |
| C400 | -0.31 | NS | -0.20 | NS | 4 | 360.2043 | 256.2642 |
| C401 | 0 | NS | -0.34 | NS | 4 | 310.8003 | 495.3288 |
| **C402** | **-1.03** | **0.0301** | -0.49 | NS | **4** | **309.5323** | **148.5328** |
| **C403** | **-2.40** | **0.0300** | -1.38 | NS | **4** | **309.291** | **1039.679** |
| C404 | 0.08 | NS | -0.19 | NS | 4 | 321.8203 | 551.3604 |
| lysoPC(18:2).3 | 0.90 | NS | -0.13 | NS | 2 | 321.606 | 520.3401 |
| C406 | 0.28 | 0.1425 | -0.11 | NS | 4 | 320.556 | 240.1004 |
| C407 | -0.66 | NS | 0.09 | NS | 4 | 336.4257 | 487.3607 |
| C408 | 0.01 | NS | -0.22 | NS | 4 | 335.4033 | 184.074 |
| C409 | 0 | NS | 0.06 | NS | 4 | 332.262 | 502.327 |
| C410 | -0.18 | NS | -0.20 | NS | 4 | 330.7477 | 280.1474 |
| C411 | -0.06 | NS | -0.24 | NS | 4 | 329.2407 | 135.1178 |
| C412 | 0.56 | NS | -0.09 | NS | 4 | 330.3107 | 302.2458 |
| FAME(18:2) | -0.31 | NS | -0.39 | NS | 2 | 325.7193 | 263.2376 |
| C414 | 0.34 | NS | 0.05 | NS | 4 | 299.0733 | 431.2472 |
| C415 | 0.10 | NS | 0.06 | NS | 4 | 301.154 | 286.1244 |
| C416 | -0.01 | NS | 0.18 | NS | 4 | 286.5733 | 239.1327 |
| C417 | -0.62 | NS | -0.32 | NS | 4 | 267.062 | 318.2407 |
| C418 | 0.13 | NS | 0.12 | NS | 4 | 253.914 | 301.142 |
| **C419** | **-1.25** | **0.0793** | -0.06 | NS | **4** | **229.378** | **389.2694** |
| C420 | -2.78 | 0.1559 | -0.36 | NS | 4 | 219.389 | 241.1741 |
| C421 | -3.33 | 0.1425 | 0.71 | NS | 4 | 209.822 | 225.1968 |
| C422 | 0.13 | NS | -0.03 | NS | 4 | 207.354 | 290.2698 |
| C423 | 0.15 | NS | 0.29 | NS | 4 | 697.4213 | 799.6721 |
| C424 | 0.22 | 0.1253 | 0.14 | NS | 4 | 725.501 | 365.1062 |
| C425 | 0.21 | NS | -0.09 | NS | 4 | 726.5533 | 647.4622 |
| C426 | 0.18 | NS | 0.17 | NS | 4 | 720.372 | 99.9697 |
| C427 | -0.77 | NS | 0.06 | NS | 4 | 603.493 | 842.5255 |
| **C428** | **0.41** | **0.0401** | -0.18 | NS | **4** | **590.0267** | **701.5606** |
| 16:0 SM (d18:1/16:0).2 | 1.30 | 0.1601 | 0.42 | NS | 2 | 590.587 | 702.568 |
| C430 | -0.24 | NS | 0.50 | NS | 4 | 582.5533 | 371.2618 |
| C431 | 0.52 | NS | 0.48 | NS | 4 | 656.2727 | 813.621 |
| C432 | 0.48 | 0.1549 | 0.09 | NS | 4 | 663.712 | 830.0752 |
| C433 | 0.67 | NS | 0.24 | NS | 4 | 664.1823 | 814.6275 |
| C434 | -0.05 | NS | -0.18 | NS | 4 | 618.792 | 319.1952 |
| 1,2-dilinoleoyl-sn-glycero-3-phosphocholine.1 | 0.04 | NS | -0.38 | NS | 1 | 621.6967 | 781.562 |
| C436 | 0.62 | 0.1658 | 0.12 | NS | 4 | 621.1337 | 830.5688 |
| C437 | 0.53 | NS | 0.07 | NS | 4 | 622.8717 | 359.1681 |
| C438 | 0.21 | NS | 0.02 | NS | 4 | 623.5857 | 852.5534 |
| C439 | -0.06 | NS | 0.54 | NS | 4 | 626.474 | 780.0575 |
| C440 | 0.20 | NS | -0.12 | NS | 4 | 651.6813 | 787.5459 |
| C441 | 0.42 | NS | 0.06 | NS | 4 | 654.0297 | 790.5746 |
| C442 | 0.03 | NS | -0.14 | NS | 4 | 652.12 | 479.3335 |
| C443 | -0.08 | NS | -0.03 | NS | 4 | 650.6073 | 798.5542 |
| C444 | -0.32 | NS | -0.25 | NS | 4 | 644.789 | 780.557 |
| C445 | 0.60 | 0.1138 | 0.08 | NS | 4 | 644.8683 | 830.5698 |
| C446 | 0.30 | NS | 0.18 | NS | 4 | 634.4423 | 784.5787 |
| C447 | 0.35 | NS | -0.24 | NS | 4 | 635.0433 | 343.195 |
| C448 | -0.07 | NS | -0.02 | NS | 4 | 632.678 | 860.5071 |
| C449 | 0.25 | NS | 0.21 | NS | 4 | 628.1683 | 365.1058 |
| C450 | 0.27 | NS | -0.31 | NS | 4 | 637.727 | 803.0531 |
| C451 | 0.52 | NS | -0.13 | NS | 4 | 637.6513 | 768.5604 |
| C452 | 0.01 | NS | -0.01 | NS | 4 | 635.6813 | 866.5362 |
| C453 | 0.28 | NS | 0.63 | NS | 4 | 637.203 | 794.6078 |
| C454 | 0 | NS | -0.35 | NS | 4 | 557.1393 | 806.5153 |
| C455 | 0.04 | NS | 0.20 | NS | 4 | 804.44 | 97.9693 |
| **C456** | **0.31** | **0.0284** | 0.14 | NS | **4** | **744.636** | **365.1059** |
| C457 | 0.04 | NS | 0.17 | NS | 4 | 907.8523 | 141.9595 |
| C458 | 0.25 | 0.1031 | -0.03 | NS | 4 | 1095.117 | 131.9511 |
| C459 | 0.10 | NS | 0.11 | NS | 4 | 1110.554 | 131.9511 |
| C460 | 0.02 | NS | 0.19 | NS | 4 | 999.303 | 97.9694 |
| C461 | 0.37 | NS | -0.11 | NS | 4 | 1078.7 | 474.8345 |
| C462 | 0.25 | NS | -0.03 | NS | 4 | 42.85333 | 235.0929 |
| C463 | 0.19 | NS | 0.05 | NS | 4 | 37.457 | 359.0438 |
| C464 | 0.21 | NS | 0.06 | NS | 4 | 35.55 | 247.0927 |
| **C465** | **-1.28** | **0.0039** | 0 | NS | **4** | **35.14667** | **197.0911** |
| **Betaine** | **-0.64** | **0.0125** | -0.02 | NS | **1** | **34.17233** | **117.079** |
| C467 | 0.19 | NS | 0.17 | NS | 4 | 30.75933 | 327.051 |
| C468 | 0.19 | NS | 0.02 | NS | 4 | 31.37233 | 310.9137 |
| C469 | 0.19 | NS | 0.13 | NS | 4 | 31.46367 | 226.889 |
| C470 | -0.01 | NS | 0.04 | NS | 4 | 31.29833 | 336.016 |
| C471 | 0.06 | NS | 0.03 | NS | 4 | 30.958 | 1099.788 |
| C472 | 0.08 | NS | -0.09 | NS | 4 | 53.86167 | 86.0606 |
| C473 | 0.02 | NS | 0.08 | NS | 4 | 67.97033 | 217.0828 |
| Phenylalanine | -0.10 | NS | 0.06 | NS | 1 | 82.55433 | 121.0846 |
| C475 | -0.11 | NS | 0.01 | NS | 4 | 119.196 | 235.0941 |
| **C476** | **-1.35** | **0.0005** | -0.07 | NS | **4** | **119.8117** | **431.0979** |
| **Phenylacetylglycine** | **-1.74** | **0.0058** | 0.17 | NS | **2** | **122.3187** | **193.074** |
| C478 | 0.15 | NS | 0.09 | NS | 4 | 117.6487 | 349.1847 |
| C479 | 0.09 | NS | 0.15 | NS | 4 | 115.2877 | 302.0206 |
| C480 | 0.02 | NS | 0.17 | NS | 4 | 137.401 | 242.2854 |
| C481 | 0.13 | NS | 0.25 | NS | 4 | 125.7093 | 673.2523 |
| **C482** | **-1.53** | **0.0075** | 0.08 | NS | **4** | **128.2837** | **307.0831** |
| C483 | -0.40 | NS | -0.01 | NS | 4 | 149.118 | 167.105 |
| C484 | -3.17 | 0.1253 | 0.56 | NS | 4 | 155.7297 | 167.9946 |
| C485 | -1.55 | NS | 0.27 | NS | 4 | 173.7387 | 212.0206 |
| C486 | -0.07 | NS | -0.17 | NS | 4 | 1314.572 | 203.0533 |
| **C487** | 0.25 | NS | **0.43** | **0.0350** | **4** | **472.369** | **203.0535** |
| C488 | -0.15 | NS | -0.25 | NS | 4 | 458.392 | 414.2703 |
| C489 | 0.41 | NS | 0.06 | NS | 4 | 410.748 | 95.0864 |
| C490 | 1.91 | NS | 0.86 | NS | 4 | 412.5965 | 337.1825 |
| C491 | 0.38 | NS | 0.40 | NS | 4 | 416.4715 | 383.2123 |
| C492 | 0.16 | NS | 0.20 | NS | 4 | 415.3165 | 226.0655 |
| C493 | 0.11 | NS | -0.03 | NS | 4 | 440.1555 | 245.2275 |
| **C494** | **0.95** | **0.0658** | 0.82 | 0.1331 | **4** | **431.919** | **368.2013** |
| C495 | 0.44 | 0.1943 | -0.23 | NS | 4 | 391.6385 | 184.0741 |
| C496 | -0.03 | NS | 0.06 | NS | 4 | 389.715 | 457.3514 |
| C497 | 1.38 | NS | -0.10 | NS | 4 | 389.4765 | 319.1952 |
| C498 | 1.28 | NS | -0.07 | NS | 4 | 387.5515 | 335.1893 |
| C499 | 0.20 | 0.1559 | 0.12 | NS | 4 | 384.595 | 140.9886 |
| **C500** | **-0.55** | **0.0989** | -0.34 | NS | **4** | **383.867** | **338.1995** |
| C501 | -0.80 | NS | -1.04 | 0.1559 | 4 | 375.84 | 510.357 |
| **C502** | **1.44** | **0.0658** | 0.17 | NS | **4** | **374.604** | **572.3719** |
| C503 | 0.48 | NS | 0.12 | NS | 4 | 398.895 | 600.3095 |
| C504 | 0.33 | NS | -0.21 | NS | 4 | 400.1805 | 561.8169 |
| C505 | 0.63 | NS | -0.05 | NS | 4 | 401.86 | 551.3286 |
| C506 | 0.11 | NS | -0.28 | NS | 4 | 342.1435 | 496.3406 |
| C507 | 0.30 | NS | 0.03 | NS | 4 | 347.517 | 572.3718 |
| C508 | -0.24 | NS | -0.05 | NS | 4 | 348.8345 | 482.3616 |
| C509 | 0.85 | 0.1683 | -0.05 | NS | 4 | 348.0385 | 570.3563 |
| C510 | 0.41 | NS | -0.14 | NS | 4 | 371.7705 | 323.227 |
| C511 | 0.55 | NS | -0.15 | NS | 4 | 351.4795 | 546.3557 |
| C512 | 0.50 | NS | 0.34 | NS | 4 | 354.9895 | 280.654 |
| C513 | 0.30 | NS | -0.05 | NS | 4 | 356.747 | 504.3449 |
| C514 | 0.29 | NS | -0.06 | NS | 4 | 355.8055 | 267.1642 |
| C515 | -0.45 | NS | -0.24 | NS | 4 | 357.6595 | 268.2644 |
| C516 | 0.27 | NS | 0 | NS | 4 | 356.6525 | 315.161 |
| C517 | -0.31 | NS | -0.14 | NS | 4 | 361.744 | 532.3907 |
| C518 | -0.13 | NS | -0.25 | NS | 4 | 362.5805 | 549.312 |
| C519 | -0.22 | NS | -0.29 | NS | 4 | 367.2405 | 522.356 |
| C520 | 0.75 | NS | 0.01 | NS | 4 | 365.2755 | 305.2643 |
| C521 | 0.68 | NS | 0.12 | NS | 4 | 365.206 | 263.2375 |
| C522 | 0.39 | NS | 0.18 | NS | 4 | 308.343 | 431.3162 |
| C523 | 0.40 | NS | -0.13 | NS | 4 | 315.306 | 276.2302 |
| C524 | 0.01 | NS | -0.06 | NS | 4 | 315.062 | 321.2402 |
| C525 | 0.34 | 0.1425 | -0.08 | NS | 4 | 321.7105 | 556.4451 |
| C526 | 1.06 | NS | 0.03 | NS | 4 | 318.186 | 542.3226 |
| C527 | -0.05 | NS | -0.07 | NS | 4 | 323.8775 | 312.1412 |
| C528 | 0.16 | NS | -0.56 | NS | 4 | 336.3205 | 503.2964 |
| C529 | -0.06 | NS | 0.04 | NS | 4 | 335.8185 | 517.3731 |
| **C530** | **-0.96** | **0.0813** | -0.52 | NS | **4** | **335.301** | **504.3438** |
| lysoPC(20:3).1 | -0.29 | NS | -0.35 | NS | 2 | 333.5695 | 546.3519 |
| C532 | 0.67 | NS | -0.08 | NS | 4 | 333.3145 | 263.2379 |
| C533 | -0.22 | NS | -0.13 | NS | 4 | 330.927 | 547.2984 |
| C534 | 0.75 | NS | -0.26 | NS | 4 | 301.0145 | 494.3241 |
| C535 | -0.20 | NS | 0.11 | NS | 4 | 300.4325 | 575.3767 |
| C536 | -0.12 | NS | 0.01 | NS | 4 | 300.365 | 327.1503 |
| C537 | 0.57 | NS | -0.45 | NS | 4 | 300.5875 | 322.2711 |
| C538 | -0.09 | NS | 0 | NS | 4 | 301.63 | 296.1322 |
| C539 | -0.39 | NS | 0.13 | NS | 4 | 293.5335 | 228.233 |
| **C540** | **-1.17** | **0.0988** | -0.44 | NS | **4** | **289.376** | **542.3252** |
| C541 | 0.07 | NS | -0.52 | NS | 4 | 284.5425 | 322.2704 |
| C542 | -0.40 | NS | -0.24 | NS | 4 | 277.317 | 280.2637 |
| C543 | -0.37 | NS | -0.16 | NS | 4 | 280.392 | 226.2175 |
| C544 | -0.48 | NS | -0.11 | NS | 4 | 279.416 | 263.2377 |
| C545 | -0.32 | NS | -0.32 | NS | 4 | 272.134 | 320.2563 |
| C546 | 0.40 | NS | -0.26 | NS | 4 | 251.185 | 338.2642 |
| C547 | 0.14 | NS | 0.05 | NS | 4 | 250.7685 | 301.1786 |
| C548 | 1.04 | NS | 1.16 | NS | 4 | 252.8615 | 464.2834 |
| C549 | -0.03 | NS | -0.28 | NS | 4 | 245.2885 | 338.2655 |
| C550 | 0.83 | NS | 0.22 | NS | 4 | 226.2045 | 334.2356 |
| C551 | -0.42 | NS | -0.20 | NS | 4 | 227.3085 | 136.0847 |
| C552 | 0.64 | NS | -0.02 | NS | 4 | 229.014 | 335.2364 |
| C553 | -0.93 | 0.1309 | 0.62 | NS | 4 | 229.5055 | 473.31 |
| C554 | 0.50 | NS | 0 | NS | 4 | 218.1835 | 334.1704 |
| C555 | 0.12 | NS | 0.29 | 0.1559 | 4 | 217.943 | 237.1161 |
| C556 | -0.32 | NS | 0.16 | NS | 4 | 214.4115 | 246.1536 |
| C557 | -0.45 | NS | -0.58 | NS | 4 | 188.97 | 213.1471 |
| C558 | 0.10 | NS | 0.06 | NS | 4 | 202.6095 | 102.092 |
| C559 | 0.25 | NS | 0.02 | NS | 4 | 201.0265 | 290.2702 |
| C560 | 0.23 | NS | 0.07 | NS | 4 | 197.338 | 274.2747 |
| C561 | 0.07 | NS | 0.05 | NS | 4 | 198.596 | 158.1547 |
| C562 | -0.39 | NS | 0.01 | NS | 4 | 194.321 | 409.1632 |
| **C563** | **1.16** | **0.0087** | 0.13 | NS | **4** | **691.8765** | **799.6692** |
| C564 | 0.22 | NS | 0.18 | NS | 4 | 693.654 | 365.1063 |
| C565 | 0.11 | NS | 0.08 | NS | 4 | 695.957 | 99.9697 |
| C566 | 0.21 | NS | 0.20 | NS | 4 | 702.252 | 365.1059 |
| **C567** | **1.08** | **0.0015** | 0.15 | NS | **4** | **699.931** | **824.6581** |
| C568 | 0.86 | 0.1076 | 0.27 | NS | 4 | 711.04 | 813.688 |
| C569 | 0.54 | NS | 0.45 | NS | 4 | 714.068 | 801.6886 |
| C570 | -0.17 | NS | 0.23 | NS | 4 | 605.0935 | 782.5702 |
| C571 | -0.34 | NS | -0.20 | NS | 4 | 602.2325 | 802.5384 |
| C572 | 0.23 | NS | 0.20 | NS | 4 | 601.078 | 365.1064 |
| **C573** | **-0.59** | **0.0284** | -0.44 | 0.1399 | **4** | **599.4455** | **752.526** |
| C574 | -0.25 | NS | 0.05 | NS | 4 | 600.407 | 730.54 |
| C575 | -0.30 | NS | 0.02 | NS | 4 | 589.688 | 452.3631 |
| C576 | 0.22 | NS | 0.29 | 0.1943 | 4 | 580.035 | 203.0532 |
| C577 | 0.20 | NS | -0.20 | NS | 4 | 658.778 | 876.5636 |
| C578 | 0.21 | NS | 0.03 | NS | 4 | 664.38 | 359.1674 |
| C579 | -0.13 | NS | -0.10 | NS | 4 | 660.372 | 815.5664 |
| C580 | -0.14 | NS | 0.03 | NS | 4 | 661.1435 | 436.2712 |
| C581 | 0.36 | NS | -0.04 | NS | 4 | 667.966 | 788.6182 |
| C582 | 0.29 | NS | 0.26 | NS | 4 | 686.612 | 365.106 |
| C583 | -0.52 | NS | 0.22 | NS | 4 | 680 | 728.5634 |
| C584 | 0.06 | NS | 0.15 | NS | 4 | 684.088 | 203.0533 |
| C585 | 0.36 | NS | -0.10 | NS | 4 | 671.647 | 812.6175 |
| C586 | 0.31 | 0.1559 | 0.33 | 0.1249 | 4 | 618.537 | 365.1058 |
| C587 | -0.77 | NS | -2.09 | NS | 4 | 615.3705 | 541.1228 |
| **C588** | **-0.86** | **0.0989** | -0.55 | NS | **4** | **619.444** | **768.5563** |
| C589 | 0.11 | NS | 0.06 | NS | 4 | 617.988 | 872.5417 |
| C590 | 0.08 | NS | 0.11 | NS | 4 | 610.0135 | 365.1058 |
| C591 | 0.53 | NS | -0.11 | NS | 4 | 624.6445 | 786.5932 |
| C592 | 0.20 | NS | -0.05 | NS | 4 | 623.783 | 184.0741 |
| C593 | -0.80 | NS | 0.42 | NS | 4 | 623.0025 | 796.5859 |
| C594 | -0.24 | NS | -0.13 | NS | 4 | 624.451 | 844.5323 |
| C595 | 0.63 | NS | 0.23 | NS | 4 | 628.196 | 832.5813 |
| C596 | 0.16 | NS | 0.27 | NS | 4 | 656.4885 | 365.1057 |
| C597 | 0.44 | NS | 0.30 | NS | 4 | 628.8515 | 856.5865 |
| C598 | 0.44 | NS | 0.05 | NS | 4 | 634.2115 | 790.558 |
| C599 | -0.02 | NS | 0.15 | NS | 4 | 632.87 | 337.1715 |
| C600 | -0.17 | NS | -0.25 | NS | 4 | 631.969 | 787.5326 |
| C601 | -0.19 | NS | -0.05 | NS | 4 | 630.8905 | 842.5254 |
| C602 | 0.36 | NS | 0.01 | NS | 4 | 642.9155 | 321.2105 |
| C603 | 0 | NS | 0.18 | NS | 4 | 640.9365 | 184.074 |
| C604 | 0.21 | NS | -0.10 | NS | 4 | 638.481 | 370.2143 |
| C605 | 0.66 | NS | -0.11 | NS | 4 | 636.201 | 321.2103 |
| C606 | -0.64 | NS | 0.27 | NS | 4 | 634.9585 | 770.6068 |
| C607 | -0.15 | NS | -0.15 | NS | 4 | 636.624 | 870.5564 |
| C608 | 0.56 | NS | 0.33 | NS | 4 | 638.6835 | 834.5988 |
| **C609** | **-0.92** | **0.0072** | -0.17 | NS | **4** | **526.4155** | **431.3894** |
| C610 | 1.86 | 0.1639 | -0.37 | NS | 4 | 568.822 | 701.5614 |
| C611 | 0.09 | NS | 0.18 | 0.1996 | 4 | 571.9775 | 203.0534 |
| C612 | 0.34 | NS | 0.05 | NS | 4 | 569.38 | 383.3678 |
| C613 | -0.36 | NS | -1.21 | NS | 4 | 557.388 | 467.1031 |
| C614 | -0.35 | NS | -0.07 | NS | 4 | 545.5 | 675.5458 |
| C615 | 0.01 | NS | 0.09 | NS | 4 | 806.574 | 84.9602 |
| C616 | 0.10 | NS | 0.13 | NS | 4 | 762.033 | 99.9698 |
| C617 | 0.20 | NS | 0.12 | NS | 4 | 773.8015 | 99.9697 |
| C618 | 0.19 | NS | 0.10 | NS | 4 | 769.0935 | 365.106 |
| C619 | 0.07 | NS | 0.12 | NS | 4 | 794.393 | 97.9695 |
| C620 | 0.04 | NS | 0.09 | NS | 4 | 785.5985 | 97.9693 |
| C621 | 0.04 | NS | 0.18 | NS | 4 | 814.727 | 97.9694 |
| C622 | 0 | NS | 0.03 | NS | 4 | 816.956 | 84.9602 |
| C623 | 0.03 | NS | -0.02 | NS | 4 | 828.226 | 84.9602 |
| C624 | 0.05 | NS | 0.17 | NS | 4 | 827.8295 | 127.9783 |
| C625 | 0 | NS | 0.03 | NS | 4 | 847.1455 | 146.9969 |
| C626 | 0.04 | NS | 0.17 | NS | 4 | 870.71 | 97.9693 |
| C627 | 0.09 | NS | 0.16 | NS | 4 | 873.885 | 113.9643 |
| C628 | 0.10 | NS | 0.19 | NS | 4 | 865.6245 | 113.9645 |
| C629 | 0.04 | NS | 0.17 | NS | 4 | 882.552 | 97.9694 |
| C630 | 0.04 | NS | 0.21 | NS | 4 | 888.9745 | 127.9785 |
| C631 | 0.06 | NS | 0.06 | NS | 4 | 925.673 | 146.997 |
| C632 | 0.14 | NS | 0.15 | NS | 4 | 927.812 | 127.9786 |
| C633 | 0.07 | NS | 0.05 | NS | 4 | 946.428 | 84.9603 |
| C634 | 0.10 | NS | 0.20 | NS | 4 | 947.2235 | 97.9694 |
| C635 | 0.02 | NS | 0.15 | NS | 4 | 964.9965 | 97.9695 |
| C636 | 0.06 | NS | 0.05 | NS | 4 | 965.1555 | 84.9603 |
| C637 | 0.02 | NS | 0.18 | NS | 4 | 987.5925 | 97.9694 |
| C638 | 0.10 | NS | 0.22 | NS | 4 | 976.0905 | 97.9694 |
| C639 | -0.01 | NS | -0.03 | NS | 4 | 975.9355 | 146.997 |
| C640 | 0.25 | NS | 0.17 | NS | 4 | 1093.425 | 96.9615 |
| C641 | 0.36 | 0.1253 | 0.06 | NS | 4 | 1093.183 | 104.9638 |
| C642 | 0.16 | NS | 0.01 | NS | 4 | 1103.089 | 131.9512 |
| C643 | 0.18 | NS | 0.11 | NS | 4 | 1124.426 | 131.9512 |
| C644 | 0.21 | NS | 0.27 | NS | 4 | 1072.104 | 230.8908 |
| C645 | -0.23 | NS | 0.23 | NS | 4 | 1083.902 | 146.9971 |
| C646 | 0.12 | NS | 0.06 | NS | 4 | 1083.295 | 116.9857 |
| C647 | 0.45 | 0.1331 | 0.01 | NS | 4 | 1078.198 | 490.8084 |
| C648 | 0.05 | NS | -0.23 | NS | 4 | 1081.97 | 125.9866 |
| C649 | -0.20 | NS | 0.05 | NS | 4 | 1061.715 | 203.0533 |
| C650 | 0.04 | NS | 0.12 | NS | 4 | 1029.293 | 97.9694 |
| C651 | 0.08 | NS | 0.07 | NS | 4 | 1026.204 | 84.9603 |
| C652 | 0.08 | NS | 0.27 | NS | 4 | 1047.843 | 97.9694 |
| C653 | 0.26 | NS | 0.16 | NS | 4 | 47.394 | 302.0206 |
| C654 | 0.34 | NS | 0.27 | NS | 4 | 44.1635 | 189.0876 |
| C655 | 0.16 | NS | -0.04 | NS | 4 | 41.413 | 682.0685 |
| C656 | 0.18 | NS | 0.09 | NS | 4 | 40.811 | 683.0681 |
| C657 | 1.58 | NS | -0.63 | NS | 4 | 33.963 | 162.1132 |
| C658 | -0.49 | NS | 0.13 | NS | 4 | 36.996 | 226.0461 |
| C659 | 0.19 | NS | 0.05 | NS | 4 | 35.6115 | 726.0318 |
| C660 | 0.08 | NS | 0.11 | NS | 4 | 36.289 | 323.0217 |
| C661 | 0.27 | NS | 0.06 | NS | 4 | 36.022 | 389.9743 |
| C662 | 0.03 | NS | 0.21 | NS | 4 | 35.2075 | 740.068 |
| C663 | -0.29 | NS | 0.16 | NS | 4 | 33.9775 | 90.0555 |
| C664 | -0.32 | NS | 0.03 | NS | 4 | 32.5845 | 220.0692 |
| C665 | 0.25 | NS | 0.10 | NS | 4 | 32.382 | 259.028 |
| **C666** | **0.52** | **0.0658** | 0.07 | NS | **4** | **33.0905** | **164.0303** |
| **C667** | **0.52** | **0.0988** | -0.05 | NS | **4** | **33.345** | **205.0603** |
| C668 | 0.20 | NS | 0.03 | NS | 4 | 31.4215 | 1112.785 |
| C669 | -0.24 | NS | 0.19 | NS | 4 | 31.573 | 214.0576 |
| C670 | -0.03 | NS | 0.08 | NS | 4 | 29.301 | 156.0776 |
| C671 | 0.09 | NS | 0.01 | NS | 4 | 30.038 | 428.894 |
| C672 | 0.30 | 0.1966 | -0.02 | NS | 4 | 56.706 | 302.02 |
| C673 | 0.17 | NS | 0.16 | NS | 4 | 65.196 | 302.0201 |
| C674 | -0.52 | NS | 0.54 | NS | 4 | 60.901 | 294.1015 |
| C675 | 0.35 | NS | 0.20 | NS | 4 | 62.329 | 235.093 |
| C676 | 0.20 | NS | 0.01 | NS | 4 | 71.9655 | 302.0201 |
| C677 | 0.15 | NS | 0 | NS | 4 | 75.6845 | 189.0878 |
| C678 | 0.11 | NS | 0.06 | NS | 4 | 89.7455 | 217.083 |
| C679 | -0.08 | NS | 0.19 | NS | 4 | 83.699 | 120.0814 |
| C680 | 0.02 | NS | -0.12 | NS | 4 | 106.827 | 146.1184 |
| C681 | -0.29 | NS | 0.12 | NS | 4 | 112.9055 | 120.0815 |
| C682 | 0.12 | NS | 0.08 | NS | 4 | 96.2135 | 235.0932 |
| Feruloylputrescine | 0.01 | NS | 0.12 | NS | 2 | 102.261 | 177.0554 |
| C684 | 0.14 | NS | 0.23 | NS | 4 | 102.234 | 160.0958 |
| C685 | 0.25 | NS | 0.07 | NS | 4 | 120.874 | 407.1903 |
| C686 | 0.19 | NS | 0.16 | NS | 4 | 121.079 | 239.1261 |
| C687 | 0.03 | NS | 0.16 | NS | 4 | 135.3265 | 302.0205 |
| C688 | 0.28 | 0.1999 | 0.16 | NS | 4 | 132.91 | 569.3194 |
| C689 | 0.18 | NS | 0.11 | NS | 4 | 130.046 | 525.2912 |
| C690 | 1.37 | NS | -0.13 | NS | 4 | 130.533 | 210.1109 |
| C691 | 0 | NS | -0.02 | NS | 4 | 130.996 | 235.0938 |
| C692 | 0.19 | NS | 0.05 | NS | 4 | 126.876 | 495.2429 |
| **C693** | **0.59** | **0.0214** | 0.29 | NS | **4** | **127.81** | **265.9852** |
| C694 | -0.45 | NS | 0.06 | NS | 4 | 159.4725 | 163.0613 |
| C695 | 0.40 | NS | -0.81 | NS | 4 | 154.2395 | 211.0949 |
| **3-Indolepropionic acid** | **-0.85** | **0.0722** | -0.08 | NS | **1** | **153.2195** | **189.079** |

ANOVA, analysis of variance; FAME, fatty acid methyl esters; FC, log_2_ fold-change; LC-MS, liquid chromatography-mass spectrometry; m/z, mass value of compound or base peak; PC, phosphatidylcholine; PF, post-farrow; SM, sphingomyelin; WN, weaning.

NS, not significant; ANOVA p-value ≥ 0.2000.

ANOVA p-value ≤ 0.1000, bolded.

^1^Unidentified compound identifier denoted as C###.

^2^Annotation confidence score (scale of 1-4) based on guidelines provided by the Metabolomics Standards Initiative (Sumner et al., 2007).
